# Disease Burden of 32 Infectious Diseases in the Netherlands, 2007-2011

**DOI:** 10.1371/journal.pone.0153106

**Published:** 2016-04-20

**Authors:** Alies van Lier, Scott A. McDonald, Martijn Bouwknegt, EPI group, Mirjam E. Kretzschmar, Arie H. Havelaar, Marie-Josée J. Mangen, Jacco Wallinga, Hester E. de Melker

**Affiliations:** 1 Centre for Infectious Disease Control, National Institute for Public Health and the Environment (RIVM), Bilthoven, The Netherlands; 2 Julius Centre for Health Sciences and Primary Care, University Medical Centre Utrecht (UMCU), Utrecht, The Netherlands; 3 Emerging Pathogens Institute, University of Florida, Gainesville, Florida, United States of America; FDA, UNITED STATES

## Abstract

**Background:**

Infectious disease burden estimates provided by a composite health measure give a balanced view of the true impact of a disease on a population, allowing the relative impact of diseases that differ in severity and mortality to be monitored over time. This article presents the first national disease burden estimates for a comprehensive set of 32 infectious diseases in the Netherlands.

**Methods and Findings:**

The average annual disease burden was computed for the period 2007–2011 for selected infectious diseases in the Netherlands using the disability-adjusted life years (DALY) measure. The pathogen- and incidence-based approach was adopted to quantify the burden due to both morbidity and premature mortality associated with all short and long-term consequences of infection. Natural history models, disease progression probabilities, disability weights, and other parameters were adapted from previous research. Annual incidence was obtained from statutory notification and other surveillance systems, which was corrected for under-ascertainment and under-reporting. The highest average annual disease burden was estimated for invasive pneumococcal disease (9444 DALYs/year; 95% uncertainty interval [UI]: 8911–9961) and influenza (8670 DALYs/year; 95% UI: 8468–8874), which represents 16% and 15% of the total burden of all 32 diseases, respectively. The remaining 30 diseases ranked by number of DALYs/year from high to low were: HIV infection, legionellosis, toxoplasmosis, chlamydia, campylobacteriosis, pertussis, tuberculosis, hepatitis C infection, Q fever, norovirus infection, salmonellosis, gonorrhoea, invasive meningococcal disease, hepatitis B infection, invasive *Haemophilus influenzae* infection, shigellosis, listeriosis, giardiasis, hepatitis A infection, infection with STEC O157, measles, cryptosporidiosis, syphilis, rabies, variant Creutzfeldt-Jakob disease, tetanus, mumps, rubella, diphtheria, and poliomyelitis. The very low burden for the latter five diseases can be attributed to the National Immunisation Programme. The average disease burden per individual varied from 0.2 (95% UI: 0.1–0.4) DALYs per 100 infections for giardiasis, to 5081 and 3581 (95% UI: 3540–3611) DALYs per 100 infections for rabies and variant Creutzfeldt-Jakob disease, respectively.

**Conclusions:**

For guiding and supporting public health policy decisions regarding the prioritisation of interventions and preventive measures, estimates of disease burden and the comparison of burden between diseases can be informative. Although the collection of disease-specific parameters and estimation of incidence is a process subject to continuous improvement, the current study established a baseline for assessing the impact of future public health initiatives.

## Introduction

Accurate estimates of the current and future disease burden of specific infectious diseases can support national public health policy. Information regarding the ranked estimated disease burden among a number of infectious diseases can guide priority setting within the field of infectious disease prevention and control. Infectious diseases and their short- and long-term consequences (i.e., complications and sequelae) are quite heterogeneous in terms of severity and the risk of mortality. Infections with certain pathogens are common but with relatively mild health consequences, whereas others may be associated with a high mortality rate, but occur only rarely. Consequently, it is difficult to compare the burden of different diseases based solely on incidence or mortality rates. Comparison of rates of disease burden within the international context can also be unduly challenging unless standardised methodologies and measures of burden are adopted [1].

To enable such comparisons, several composite health measures have been developed that combine morbidity and mortality [2], such as the disability-adjusted life year (DALY) measure developed for the Global Burden of Diseases, Injuries, and Risk Factors study (GBD) [3–5]. The idea behind the DALY approach is that the impact of a particular disease can be divided into the number of years of life lost (i.e., premature mortality) and the number of years lived at less than full health (i.e., morbidity). The result is a single measurement unit that quantifies the years of healthy life lost due to a certain disease or infection. The DALY has since been widely applied for estimating disease burden at national, regional, and global levels [5–9].

The current study constitutes the largest study, in terms of number of infectious diseases, in Europe on the disease burden of infectious diseases that is derived using national data sources. Methodological issues that we encountered in estimating the burden for the Netherlands could be of relevance to other countries who intend to calculate their national infectious disease burden. In our study, we compute national disease burden estimates expressed in DALYs for 32 infectious diseases in the Netherlands in the period 2007–2011, mainly using methodology and software from the Burden of Communicable Diseases in Europe (BCoDE) project [2, 10].

## Methods

The following diseases were included in this study a) sexually transmitted infections: chlamydia, gonorrhoea, hepatitis B infection, hepatitis C infection, HIV infection, and syphilis, b) vaccine-preventable diseases: diphtheria, invasive *Haemophilus influenzae* infection, invasive meningococcal disease, invasive pneumococcal disease, measles, mumps, pertussis, poliomyelitis, rabies, rubella, and tetanus, c) food-related diseases: campylobacteriosis, cryptosporidiosis, giardiasis, hepatitis A infection, listeriosis, norovirus infection, salmonellosis, shigellosis, toxoplasmosis, variant Creutzfeldt-Jakob disease, and infection with STEC O157, d) respiratory diseases: influenza, legionellosis, Q fever, and tuberculosis. This set of 32 diseases was selected based on diseases included in the BCoDE project [10, 11]. We excluded tick-borne encephalitis from the BCoDE list because it is not endemic in the Netherlands, and we added norovirus infection as an additional pathogen that is known to cause considerable disease burden [12].

### Computation of disability-adjusted life-years (DALY)

The DALY is the simple sum of two components: (1) premature mortality, quantified as the number of years of life lost (Years of Life Lost = YLL), and (2) morbidity, the number of healthy years lost due to that health outcome (Years Lived with Disability = YLD). The DALY for a pathogen is therefore the sum of the YLL and YLD associated with all health outcomes that can be attributed to the infection by that pathogen.

YLL is calculated as the number of deaths (estimated from the case-fatality rate and the number of cases) multiplied by the remaining life expectancy at the age of death, summed over all fatal health outcomes within the natural history of the disease, with respect to a reference population and time period (normally describing an ideal, or maximum life expectancy) [9, 13]. YLD is calculated by multiplying per health outcome the number of incident cases for that outcome by the disability weight—a measure of the severity of the health outcome/disabling condition—and by the duration of the health outcome, summed over all health outcomes comprising the natural history for that disease, in a given population and time period. All parameters for both YLL and YLD can be specified by age and/or sex.

Several fundamental methodological decisions are required for burden of disease estimation [10]. We followed the BCoDE protocol and used the pathogen-based approach [9, 10], in which the focus of burden calculation is on all health outcomes that can be causally attributed to that specific pathogen. This approach gives justice to the potential long-term sequelae of infectious diseases, and permits a better estimation of the (future) health benefits associated with the prevention of infections.

We calculated disease burden based on incidence data [9, 10]. In this way, all new cases of a particular disease are counted, and the burden associated with all (future) health outcomes is included, which is assigned to the year of initial infection. Working with incidence data can lead to a better understanding of the possible future health gains from prevention initiatives that decrease the incidence of infection. However, the incidence approach does not take into account the burden of disease among patients who have contracted a [chronic] infectious disease in the past, and still suffer from the health consequences (e.g., HIV, hepatitis B infection). Also, it is more difficult to correct for comorbidities [8].

The incidence in the period 2007–2011 (see section 2.1.4) was mainly derived through statutory notification data. For non-notifiable diseases, we located the best alternative data source(s); for instance, laboratory surveillance and sentinel general practice/primary health care surveillance systems.

We did not model treatment effects explicitly, although the effect of treatment is reflected in the national history of a disease, i.e., severity, duration, and the risk of complications or dying are all influenced by available treatment. However, when treatment options have recently changed (such as for hepatitis C infection) available parameters no longer reflect the current situation well. Such parameters will need to be updated when more information becomes available.

#### Under-estimation of incidence

It is important to establish whether data on new cases of disease, used for disease burden estimation, adequately reflect the actual situation, or additional adjustment for under-ascertainment and/or under-reporting is needed [11, 14]. *Under-ascertainment* refers to the extent to which incidence is under-estimated because there are symptomatic cases in the community who do not get in contact with health services, such as their general practitioner. They may have no contact because they suffer from mild illness only and/or symptoms are self-limiting. *Under-reporting* refers to those infected individuals who do contact health services, but whose disease status is incorrectly diagnosed or classified, or fails to be reported to the organisation responsible for surveillance.

Appropriate multiplication factors (MFs)–with uncertainty intervals if available–were derived by disease surveillance specialists. For a number of pathogens, there was sufficient information to specify age- and/or sex-dependent MFs. For others, a single MF–either a point estimate or a range, depending on the information available–was applied for both sexes and all age-groups. Multiplication factors were chosen to either adjust in one step (under-estimation), or in two steps (under-reporting and under-ascertainment) (see S1 Appendix). The multiplication factors were based either on published studies or from analyses of relevant datasets, or on a combination of the two. Additionally, for invasive pneumococcal disease, campylobacteriosis, and salmonellosis, correction of the reported case numbers for the coverage of the surveillance system needed to be applied because some systems do not cover the whole Dutch population. In the case of HIV infection, we did not attempt to back-calculate incidence from data on new HIV diagnoses in care that constitutes routine HIV surveillance in the Netherlands; instead, burden was computed from annual new HIV diagnoses, adjusted for reporting delay.

#### Life expectancy, disability weights and durations

The same life expectancy values were adopted both for the calculation of YLL and for YLD (i.e., for long-term sequelae that persist until death) and, as in earlier GBD studies, were derived from a standard life table (West Level 26) [3, 15].

The YLD for a given health outcome is weighted for the severity of illness using disability weights. A disability weight can range from 0 (perfect health) to 1 (death) and is typically based on the preferences of a panel (of patients, medical experts or lay people) that rates the relative undesirability of hypothetical health outcomes. We adopted a set of disability weights compiled from several sources; these weights were derived using a mix of Person-Trade-Off and more novel techniques [16], similar to methods used by the original GBD [5] and other disease burden assessments [17].

Disability durations for each health outcome, required for the calculation of YLD, were based on literature review and/or expert opinion. For more details on the life expectancy, disability weights and durations we used, see State of Infectious Diseases in the Netherlands, 2013 [18, 19] and online appendix at http://www.rivm.nl/bibliotheek/rapporten/appendix150205001.pdf.

#### Outcome trees

The pathogen-based approach requires outcome trees for each pathogen, which describe the various health outcomes (including sequelae and death) following infection and how they are related within a disease’s natural history [9–11]. The health outcome ‘asymptomatic infection’ does not contribute to the disease burden, but may lead to symptomatic cases or sequelae later in life (e.g., hepatitis B infection). For several pathogens, it was necessary to divide a single health outcome into multiple ‘health states’ because of differences in severity. Pathogen outcome trees (including transition probabilities) developed as part of the BCoDE project were adapted to better fit the Dutch context (see State of Infectious Diseases in the Netherlands, 2013 [18, 19], and for the exact parameter values used, the online appendix available at http://www.rivm.nl/bibliotheek/rapporten/appendix150205001.pdf).

For nine of the eleven diseases caused by possible food-related pathogens we used the outcome trees and disease models developed by Havelaar *et al*. [12, 20]. The latter methodology is designed specifically for estimating the burden of food-related pathogens in the Netherlands. Given that not all cases caused by these pathogens are foodborne, and exposure may have occurred through other transmission routes, we term these cases food-related for conciseness.

#### Other requirements for disease burden computation

Incidence data for most pathogens were stratified by sex and by 5-year age-group (<1 years, 1–4 years, 5–9 years, …, 80–84 years, 85+ years). However, for most food-related diseases (other than shigellosis, listeriosis, toxoplasmosis, hepatitis A infection, and variant Creutzfeldt-Jakob disease), six different age-groups were used: <1 years, 1–4 years, 5–11 years, 12–17 years, 18–64 years and 65+ years. For some diseases (see Table 1), cases with unknown age and/or sex were imputed using the univariate method, and for influenza only, for which the sex distribution of cases was unknown, the sex distribution of the total population was assumed to apply.

**Table 1:** Total number of *new* cases in 2007–2011 in the Netherlands, multiplication factors (MFs) chosen to adjust for under-estimation, and the estimated annual number of total infections and deaths (averaged over the period 2007–2011 and adjusted for under-estimation and the proportion symptomatic), per disease.

Disease	Total number of new cases					MF(s) chosen (see S1 Appendix)	Estimated mean annual number 2007–2011	
	2007	2008	2009	2010	2011		Infections	Deaths
**Sexually-transmitted infections**								
Chlamydia (a)	35658	37053	35930	33882	35765	UR: 1.111	181481	0.002
Gonorrhoea *	1830	1969	2426	2815	3578	UE: 2.53	9195	0.03
Hepatitis B infection	227	219	208	197	159	UA: 1.33	1124	14
						UR: Uniform(1.20,1.22)		
Hepatitis C infection (e)	44	45	52	47	68	UE: Uniform(1, 5.12)*29/30 + Pert(0, 47, 464.4)*1/30 (d)	1233	8
HIV infection (b)	1194	1246	1134	1093	855	UE: 1	1922	115
Syphilis * (c)	660	793	711	696	545	UE: 4.21	5761	0.4
**Vaccine-preventable diseases**								
Diphtheria	0	0	0	0	0	-	0	0
Invasive *H*. *influenzae* infection *	115	108	129	143	139	UE: Uniform(1.05,1.20)	143	11
Invasive meningococcal disease *	186	159	157	137	99	UE: 1.05	155	16
Invasive pneumococcal disease (f)	2648	2328	2408	2252	2496	UE: Uniform(1.05,1.20)	2729	410
Measles	10	109	15	15	50	UE: Uniform(11.11,14.93)	518	2
Mumps *	n/a	n/a	32	424	642	UA: 1.84	673	0.005
						UR: 1		
Pertussis *	7374	8745	6461	3733	5450	UE: 21.9 (0–9 yrs); 25.0 (>9 yrs)	155480	29
Poliomyelitis	0	0	0	0	0	-	0	0
Rabies	0	1	0	0	0	UE: 1	0.2	0.2
Rubella (j)	1	2	7	0	1	UE: Uniform(11.11,14.93)	29	0.002
Tetanus	n/a	n/a	1	1	6	UE: Uniform(1.0,1.41)	3	0.3
**Food-related diseases**								
Campylobacteriosis (d,f)	6731	6431	7256	8294	8547	UE: 13	95420	39
Cryptosporidiosis (d,g)	184	184	184	184	184	[12, 20]	28100	2
Giardiasis (d,h)	2331	2142	1982	1821	1658	[12, 20]	78960	2
Hepatitis A infection (d)	168	183	176	262	125	UE: 5	894	3
Listeriosis (d)	66	52	79	77	88	UE: 1	72	5
- perinatal	6	1	3	4	9		5	1
- acquired	60	51	76	73	79		68	4
Norovirus infection (d)	n.a.	n.a.	n.a.	n.a.	n.a.	[12, 20]	655100	60
Salmonellosis (d,f)	1968	2576	1921	2291	2029	UE: 18	38820	40
Shigellosis (i)	389	438	411	522	577	UE: PERT(1.2,11.6,49.6)	7561	1
- Toxoplasmosis (d)	n.a.	n.a.	n.a.	n.a.	n.a.	[12, 20]	795	13
- congenital							371	13
- acquired							424	0
vCreutzfeldt-Jakob disease	0	0	1	0	0	UE: 1	0.2	0.2
Infection with STEC O157 (d)	83	45	57	51	65	UE: 35	2128	4
**Respiratory diseases**								
Influenza **	39028	73455	135170	18390	92887	UA: Uniform(4.12,5.13)	331995	432
						UR: 1		
Legionellosis	322	337	252	467	312	UA: 1	4407	176
						UR: PERT(9.95,11.03,24.14)		
Q fever	168	1000	2354	504	81	UE: PERT(0.75,1.575,3.25) (0–14 yrs); PERT(2.4,5.04,10.4) (15+ yrs)	11271	18
Tuberculosis *	999	1013	1158	1068	1003	UA: 1	16295	60
						UR: Uniform(1.08,1.16)		

UE = under-estimation, UA = under-ascertainment, UR = under-reporting, n/a = not available for indicated years, n.a. = not applicable, because incidence was based on seroprevalence and other studies rather than national reported figures.

*Notes*:

* Cases with unknown age and/or sex were imputed using the univariate method.

** Because the sex distribution of cases was unknown, we applied the sex distribution of the total population.

(a) The total number of cases at STI centres was only available for 2010, and so was assumed to apply to other years as well. Reported cases for 2007 are assumed to be the same as the number of cases averaged over 2008–2011, as the number of cases at sentinel general practitioners was unavailable for 2007.

(b) Based on new HIV diagnoses in care. The estimated annual number of cases also reflects adjustment for reporting delay.

(c) Cases consist of both acquired and congenital syphilis.

(d) For these food-related diseases, a different estimation method was used, see Havelaar *et al*., 2012 [12, 20].

(e) MF is a weighted sum derived from the estimated incidences of HCV among HIV-positive and HIV-negative MSM, weighted for the proportion of notified cases represented by the two respective groups. Note that the estimated annual incidence is quite uncertain (95% CI: 855–1662); this is due to the wide MF distribution specified for HIV-negative MSM, itself attributable to the wide uncertainty range in the incidence rate estimated for this group. This MF was only applied to males aged 20–69 years; for all other age groups and females, MF was set to 1.

(f) Corrected for coverage of the sentinel surveillance system: 25% coverage for invasive pneumococcal disease, 52% coverage for campylobacteriosis, and 64% coverage for salmonellosis.

(g) Calculated from the reported incidence rate for 2007; a constant incidence from 2007 onwards was assumed.

(h) Calculated from a linear regression model fitted to the reported incidence rate between 2001–2007.

(i) Total notified cases for 2011 includes 161 cases that were not culture-confirmed and perhaps should have not been included; this was due to the sudden popularity of PCR testing and culture-confirmation in 2011–12. Culture-confirmation has been legally required since 2013.

(j) Cases consist of acquired rubella only; no cases of congenital rubella syndrome were reported.

The incidence of a given infectious disease may fluctuate greatly from year to year due to infection attack rates that vary across seasons (e.g., influenza), or because of build-up of a pool of susceptibles over years (e.g., measles in the Netherlands). As a result, the estimated disease burden for a given year may not be representative of the ‘typical’ burden associated with the pathogen. As a partial solution to this issue, we estimated the mean annual incidence over a five-year period (2007–2011, the most recent five year period for which data was available) to smooth temporal fluctuations. However, in the presence of an increasing or decreasing temporal trend, this may lead to under- or over-estimation, respectively, of the current number of infectious and associated disease burden. For diseases exhibiting outbreak years (e.g., measles, pertussis, rubella, influenza, and Q fever), we discuss the magnitude of the impact of an outbreak year on our estimates.

Finally, we chose not to implement age-weighting and discounting [3], in agreement with GBD 2010 methods [21].

### Software for burden estimation

We used version 0.94 of the BCoDE software toolkit [22] (which implements the incidence- and pathogen-based approached to disease burden estimation) to estimate the burden for 23 diseases (i.e., excluding campylobacteriosis, cryptosporidiosis, giardiasis, hepatitis A infection, listeriosis, norovirus infection, salmonellosis, toxoplasmosis, and infection with STEC O157, for which the Monte Carlo simulations conducted by Havelaar *et al*. [12, 20] and implemented in Analytica software were used). An updated version of the toolkit is available for download at http://ecdc.europa.eu/en/healthtopics/burden_of_communicable_diseases/Pages/Tool.aspx.

For all diseases, but especially relevant for diseases with long-term sequelae, ageing of the infected ‘cohort’ was taken into the account by summing the duration(s) spent in previous health outcomes, so that the relevant age-group specific transition probabilities and/or case-fatality rates would be applied correctly at all points in the disease natural history.

Uncertainty intervals around mean DALYs and other outputs were estimated using Monte-Carlo sampling methods; 5000 iterations were run per disease model (7000 for each of the nine diseases in Analytica). Specifically, for transition probabilities and multiplication factors specified as distributions (Uniform or PERT; the latter is a special case of the Beta distribution specified by three parameters: a minimum, most likely, and maximum value, with a fourth scale parameter fixed at 4), the mean and 95% uncertainty interval were computed from the output distribution. For the cases in which a constant multiplication factor was provided, uncertainty was obtained using Bayesian methods that captures the intuition that there is greater uncertainty associated with low compared with high counts. In this method, the annual number of incident infections (no. cases x MF) was assumed to arise from a Poisson distribution. Therefore, the Gamma distribution (the conjugate prior for the rate parameter of the Poisson distribution), defined according to the annual incidence can be directly applied to represent uncertainty in the incidence rate [20, 23].

## Results

The total number of reported cases per year, the selected multiplication factors, and the estimated mean annual incident cases and deaths over the period 2007–2011 for all 32 diseases are provided in Table 1. Table 2 gives a comprehensive overview of the national disease burden estimates for the Netherlands per pathogen in mean YLD/year, YLL/year, DALYs/year, and DALYs per 100 infections with 95% uncertainty intervals. In the following sections, we present the results grouped into four categories: sexually-transmitted infections (including hepatitis C infection because nowadays the most frequent mode of transmission is among men who have sex with men (MSM), and hepatitis B, although also vaccine-preventable), vaccine-preventable diseases, food-related diseases, and respiratory diseases.

**Table 2:** Estimated annual disease burden in the period 2007–2011 for *new* cases of sexually-transmitted infections, vaccine-preventable diseases, food-related diseases, and respiratory diseases: mean (with 95% uncertainty intervals) YLD/year, YLL/year, DALYs/year, and DALYs/100 infections.

Disease	YLD/year	YLL/year	DALYs/year	DALYs/100 infections
**Sexually-transmitted infections**				
Chlamydia	3551	0.1	3551	2.0
	(1470–7327)	(0.1–0.2)	(1470–7328)	(0.8–4.0)
Gonorrhoea	1269	2.0	1271	14
	(666–2320)	(1.3–3.1)	(668–2323)	(7–25)
Hepatitis B infection	268	241	509	45
	(267–270)	(212–269)	(480–538)	(43–48)
Hepatitis C infection	2209	65	2274	184
	(1536–3026)	(45–95)	(1600–3085)	(130–250)
HIV infection	3811	3176	6987	363
	(3461–4175)	(2889–3476)	(6374–7622)	(332–396)
Syphilis	13	14	26	0.5
	(9–17)	(10–18)	(20–35)	(0.3–0.6)
**Vaccine-preventable diseases**				
Diphtheria	0	0	0	n.a.
Invasive	103	337	439	308
*H*. *influenzae* infection	(93–112)	(316–358)	(415–464)	(292–325)
Invasive	77	988	1065	686
meningococcal disease	(64–91)	(823–1159)	(889–1250)	(638–733)
Invasive	148	9296	9444	346
pneumococcal disease	(146–150)	(8767–9811)	(8911–9961)	(327–365)
Measles	12	119	130	25
	(11–13)	(91–145)	(103–157)	(20–30)
Mumps	3.4	0.3	3.7	0.5
	(3.1–3.6)	(0.2–0.4)	(3.4–4.0)	(0.5–0.6)
Pertussis	1633	1602	3235	2.1
	(1625–1641)	(1593–1610)	(3219–3251)	(2.1–2.1)
Poliomyelitis	0	0	0	n.a.
Rabies	0.01	10	10	5081
	(0.01–0.01)	(10–10)	(10–10)	(5081–5081)
Rubella	0.04	0.10	0.14	0.5
	(0.03–0.04)	(0.08–0.12)	(0.12–0.16)	(0.4–0.5)
Tetanus	0.07	4.3	4.4	137
	(0.07–0.08)	(3.9–4.7)	(4.0–4.8)	(132–143)
**Food-related diseases**				
Campylobacteriosis (a)	2780	534	3314	3.5
	(864–6274)	(333–809)	(1286–6872)	(2.4–7.4)
Cryptosporidiosis (a)	53	22	75	0.3
	(30–83)	(0.4–99)	(38–155)	(0.1–0.7)
Giardiasis (a)	121	29	150	0.2
	(65–206)	(0.7–117)	(78–263)	(0.1–0.4)
Hepatitis A infection (a)	53	95	148	17
	(37–83)	(57–158)	(96–237)	(13–21)
Listeriosis (a)	50	109	158	219
	(29–73)	(109–109)	(137–182)	(195–246)
- perinatal	33	81	114	2482
	(17–51)	(81–81)	(98–132)	(2128–2862)
- acquired	17	27	44	65
	(12–22)	(12–22)	(39–50)	(59–73)
Norovirus infection (a)	318	1329	1647	0.3
	(209–470)	(588–2461)	(900–2783)	(0.1–0.4)
Salmonellosis (a)	913	462	1375	3.5
	(238–2456)	(402–526)	(671–2877)	(2.3–10.9)
Shigellosis	163	33	196	2.6
	(131–198)	(26–40)	(158–236)	(2.5–2.7)
Toxoplasmosis (a)	2534	1059	3593	452
	(1114–4725)	(600–1825)	(1715–6601)	(383–583)
- congenital	1192	1059	2251	607
	(485–2449)	(681–1906)	(1088–4322)	(450–942)
- acquired	1342	0	1342	317
	(630–2276)		(627–2279)	(317–317)
vCreutzfeldt-Jakob disease	0.2	7.0	7.2	3581
	(0.1–0.3)	(6.8–7.1)	(7.1–7.2)	(3540–3611)
Infection with STEC O157 (a)	23	115	138	6.5
	(13–37)	(67–212)	(80–250)	(1.5–65)
**Respiratory diseases**				
Influenza	4090	4580	8670	2.6
	(3993–4187)	(4474–4687)	(8468–8874)	(2.6–2.6)
Legionellosis	391	3892	4283	97
	(351–435)	(3447–4389)	(3819–4805)	(90–105)
Q fever	1568	574	2143	19
	(1386–1755)	(508–642)	(1897–2395)	(17–21)
Tuberculosis	126	2615	2741	17
	(121–130)	(2117–3138)	(2241–3264)	(14–20)

(a) Burden estimated using the methods of Havelaar *et al*. [12, 20].

### Sexually-transmitted infections

**Fig 1:**
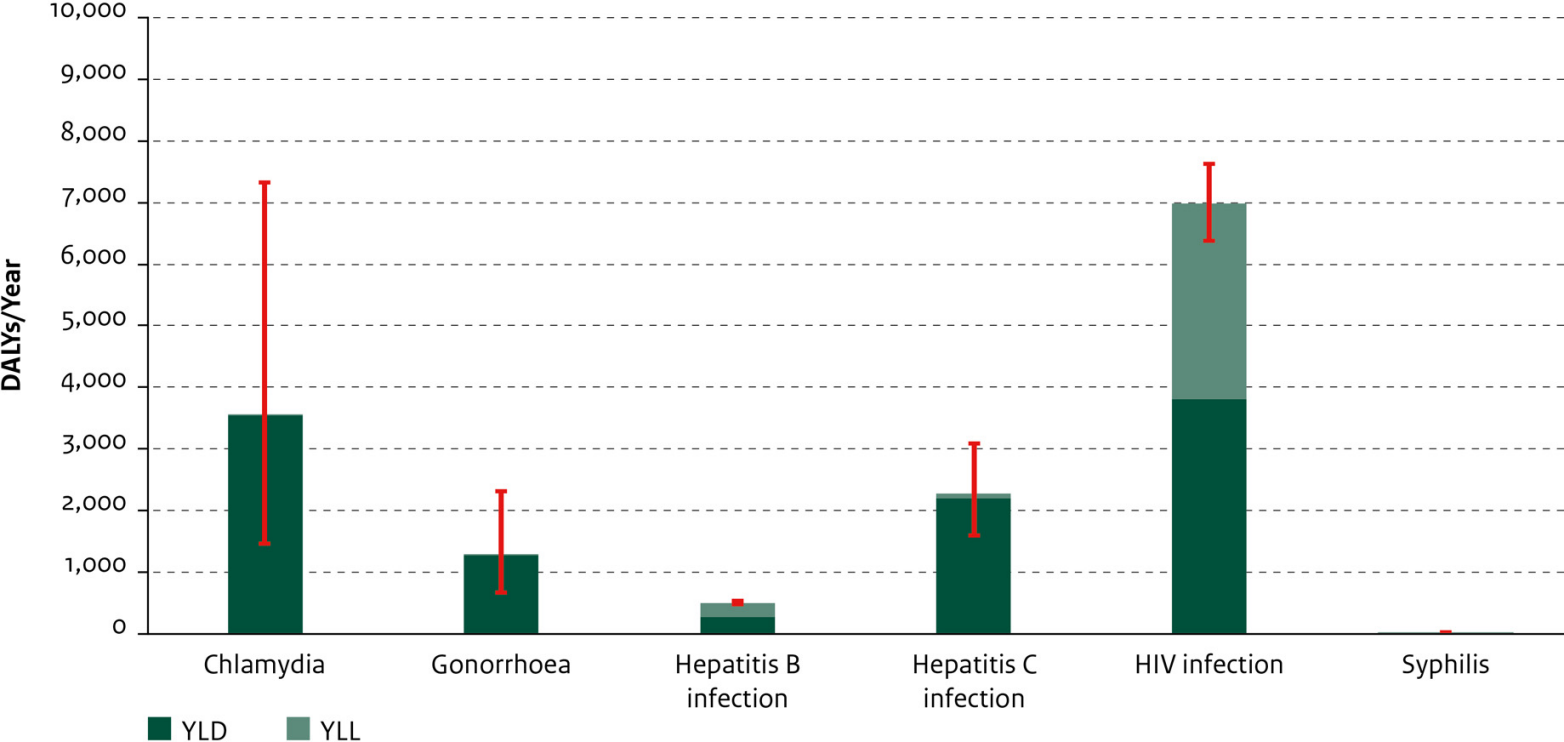
Average annual disease burden in the Netherlands in 2007–2011 for new cases of sexually-transmitted infections. YLD and YLL components are shown separately. Red lines indicate 95% uncertainty intervals.(DALY = Disability-Adjusted Life Year, YLD = Years Lived with Disability, YLL = Years of Life Lost).

**Fig 2:**
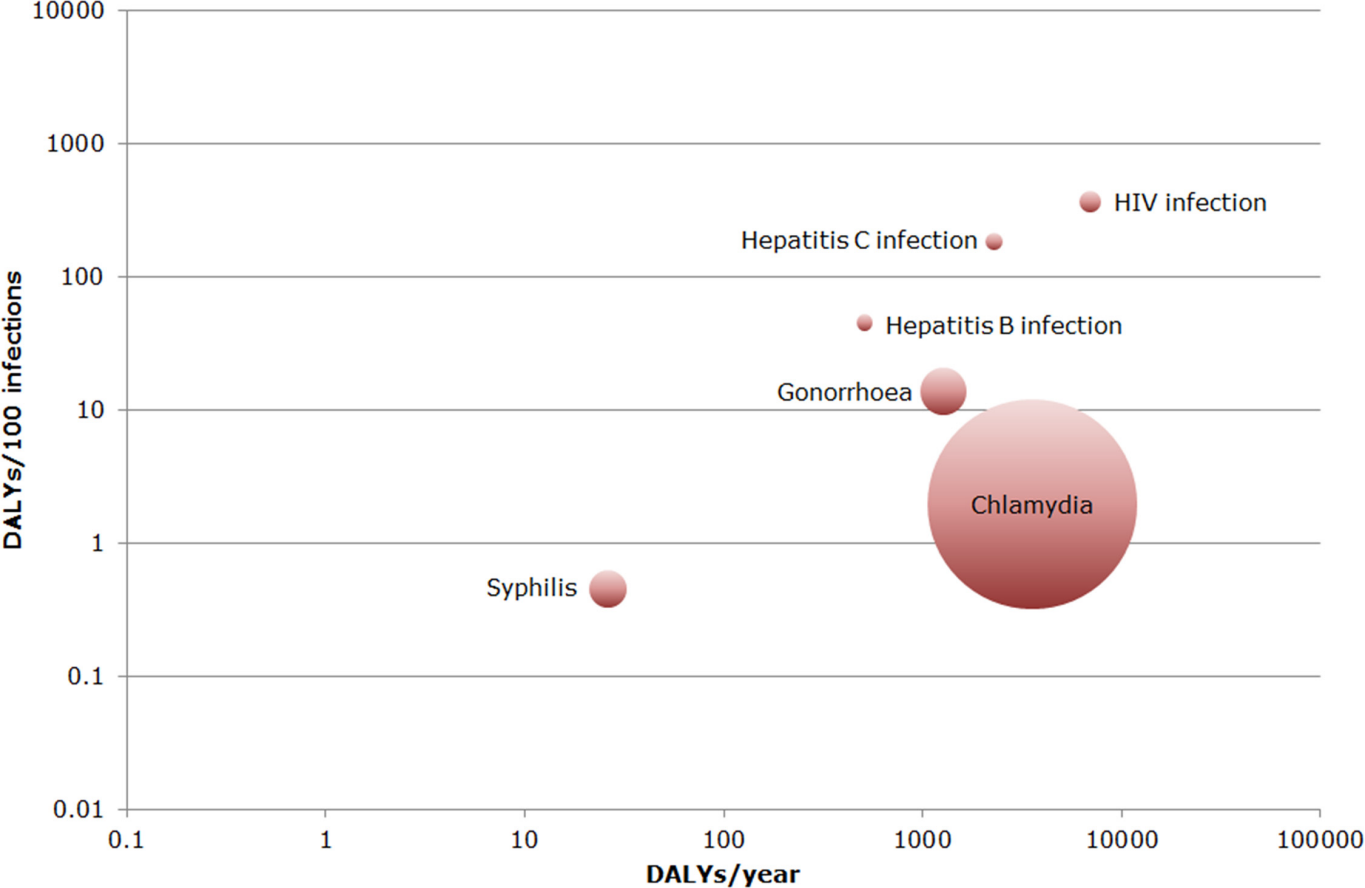
Ranking of sexually-transmitted infections by disease burden at population/individual level in the Netherlands in 2007–2011. The area of each bubble is proportional to the average number of estimated annual cases. Both axes are on a logarithmic scale.(DALY = Disability-Adjusted Life Years, population level = number of DALYs/year, individual level = number of DALYs/100 infections).

The greatest disease burden within this disease group was estimated for HIV infection (6987 DALYs/year, 95% UI: 6374–7622; largely driven by high mortality: 115 estimated deaths per year and 3176 YLL/year, 95% UI: 2889–3476), followed by chlamydia (3551 DALYs/year; 95% UI: 1470–7328), hepatitis C infection (2274 DALYs/year; 95% UI: 1600–3085), and gonorrhoea (1271 DALYs/year; 95% UI: 668–2323) (Fig 1). Syphilis had a relatively low disease burden at both the population (26 DALYs/year; 95% UI: 20–35) and the individual (0.5 DALYs/100 infections; 95% UI: 0.3–0.6) level. The other sexually-transmitted infections included had a relatively high population-level disease burden, but for chlamydia and gonorrhoea the average disease burden at the individual level was limited compared with HIV, hepatitis B and hepatitis C infection (Fig 2).

### Vaccine-preventable diseases

**Fig 3:**
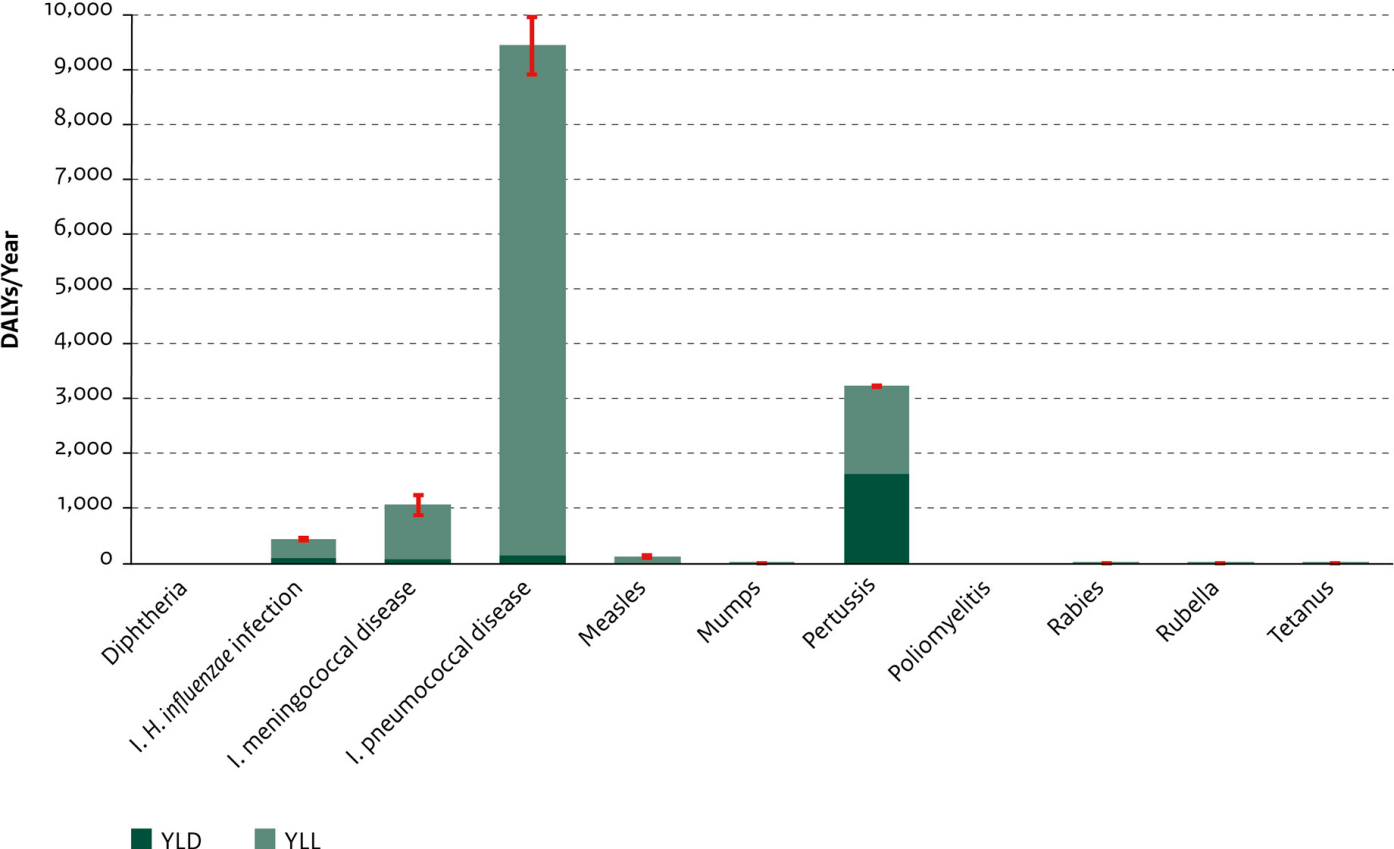
Average annual disease burden in the Netherlands in 2007–2011 for new cases of vaccine-preventable diseases. YLD and YLL components are shown separately. Red lines indicate 95% uncertainty intervals. (DALY = Disability-Adjusted Life Year, YLD = Years Lived with Disability, YLL = Years of Life Lost, I. = invasive).

For diphtheria and poliomyelitis, the estimated disease burden mounted to zero DALY because there were no incident cases reported in this period. For mumps, rabies, rubella, and tetanus, the disease burden was estimated to be very low (≤10 DALYs/year). Among vaccine-preventable diseases, the highest burden was estimated for invasive pneumococcal disease (9444 DALYs/year, 95% UI: 8911–9961; reflecting the large impact of mortality: 410 estimated deaths per year and 9296 YLL/year, 95% UI: 8767–9811), followed by pertussis (3235 DALYs/year; 95% UI: 3219–3251), and invasive meningococcal disease (1065 DALYs/year; 95% UI: 889–1250) (Fig 3). Differences in the age-distribution of disease burden were apparent; for instance, the majority of the invasive meningococcal disease burden (55%) was carried by children younger than five years of age (see Figure H in S2 Appendix).

**Fig 4:**
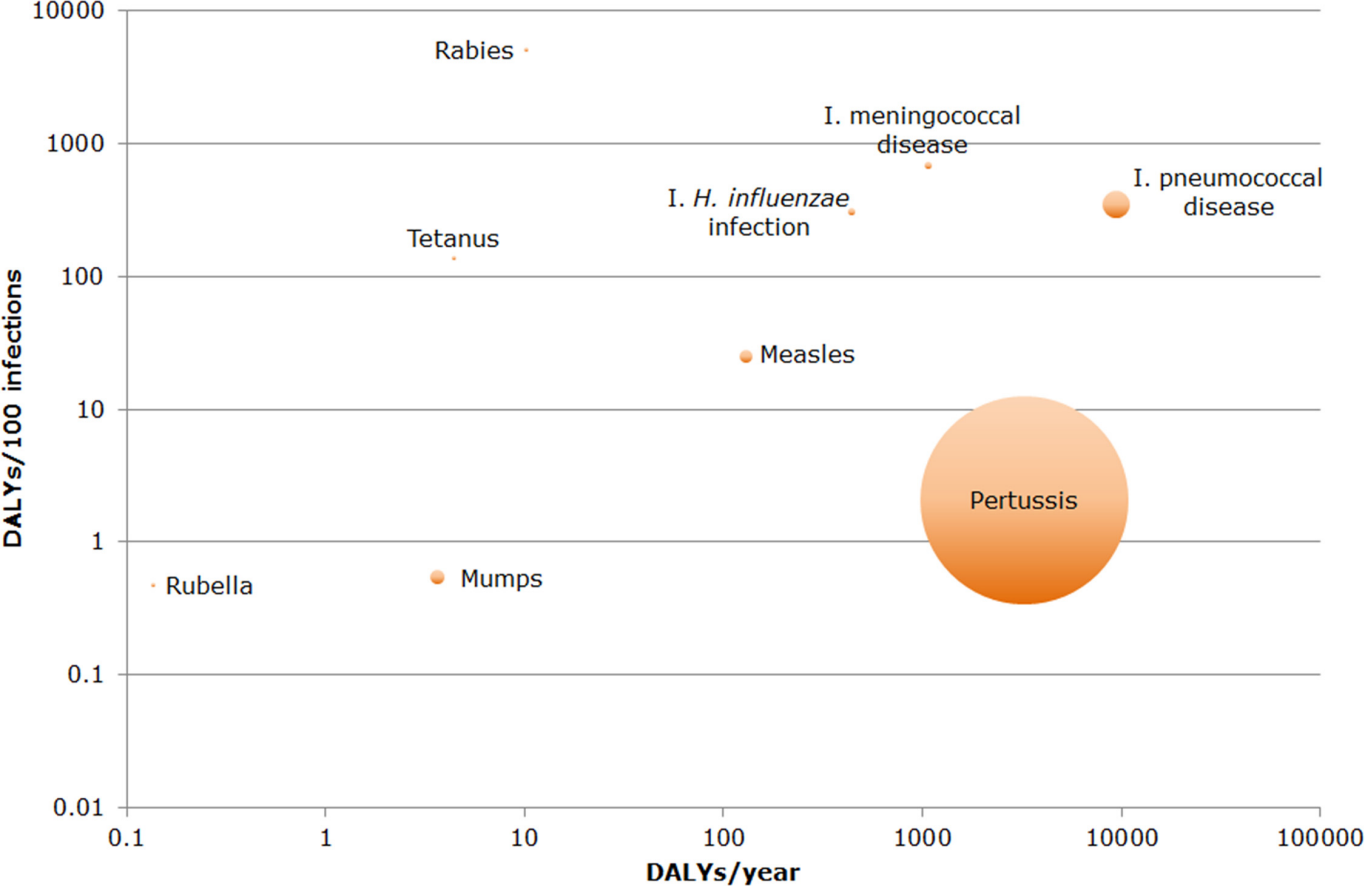
Ranking of vaccine-preventable diseases by disease burden at population/individual level in the Netherlands in 2007–2011. Diphtheria and poliomyelitis could not be included because there were no cases reported in this period. The area of each bubble is proportional to the average number of estimated annual cases (50 cases were added to each bubble for visibility reasons). Both axes are on a logarithmic scale. (DALY = Disability-Adjusted Life Years, population level = number of DALYs/year, individual level = number of DALYs/100 infections, I. = invasive).

Of the four vaccine-preventable diseases with the lowest estimated disease burden at the population level (rubella, mumps, rabies and tetanus), the average burden at the individual level for the former two diseases was low in comparison to the latter two diseases (Fig 4). Note that in this period there were no reported cases of *congenital* rubella syndrome (CRS), which has a high individual level disease burden. Among the vaccine-preventable diseases with a high estimated disease burden at the population level, the average individual-level disease burden was also quite high (with the exception of pertussis).

### Food-related diseases

**Fig 5:**
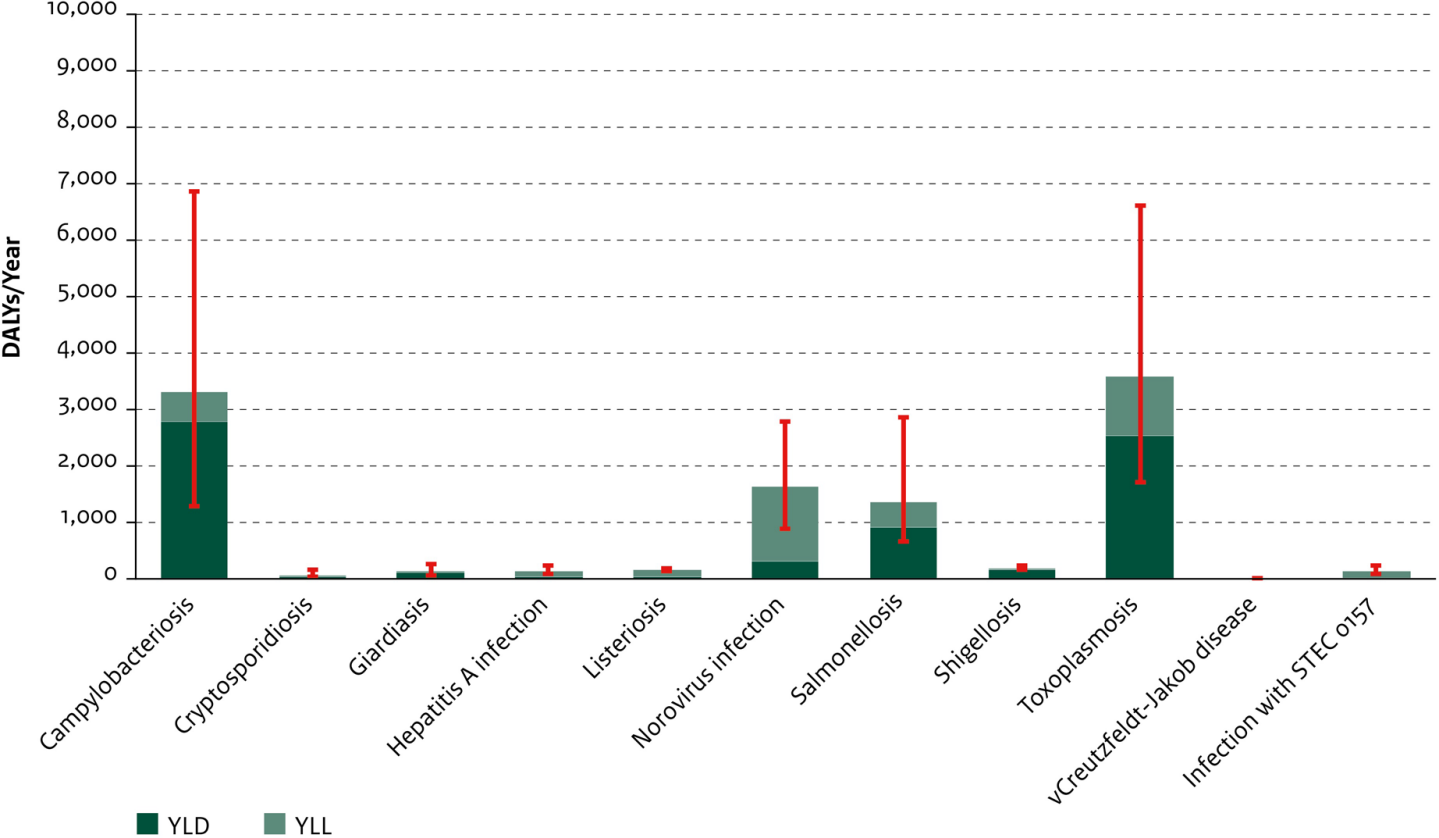
Average annual disease burden in the Netherlands in 2007–2011 for new cases of food-related diseases. YLD and YLL components are shown separately. Red lines indicate 95% uncertainty intervals. (DALY = Disability-Adjusted Life Year, YLD = Years Lived with Disability, YLL = Years of Life Lost).

**Fig 6:**
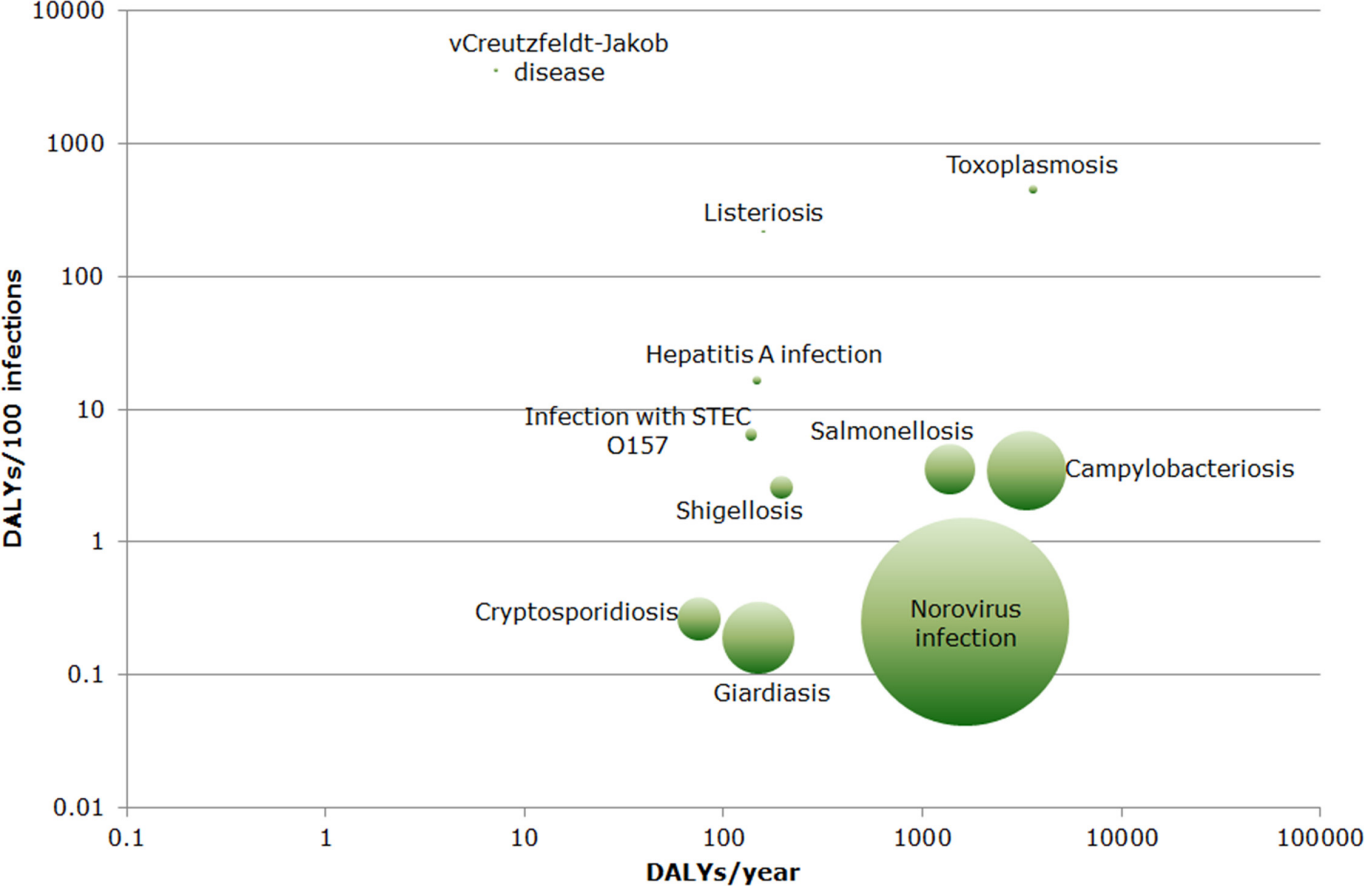
Ranking of food-related diseases by disease burden at population/individual level in the Netherlands in 2007–2011. The area of each bubble is proportional to the average number of estimated annual cases (200 cases were added to each bubble for visibility reasons). Both axes are on a logarithmic scale. (DALY = Disability-Adjusted Life Years, population level = number of DALYs/year, individual level = number of DALYs/100 infections).

The greatest disease burden at population level within this disease group was estimated for toxoplasmosis (3593 DALYs/year; 95% UI: 1715–6601), campylobacteriosis (3314 DALYs/year; 95% UI: 1286–6872), norovirus infection (1647 DALYs/year; 95% UI: 900–2783), and salmonellosis (1375 DALYs/year; 95% UI: 671–2877) (Fig 5). For most food-related diseases, the YLL component was relatively small, and the average disease burden at the individual level was low (Fig 6). Among the diseases with a high average disease burden at the individual level (i.e., variant Creutzfeldt-Jakob disease, toxoplasmosis, and listeriosis), the disease burden at the population level was comparatively limited (with the exception of toxoplasmosis).

### Respiratory diseases

**Fig 7:**
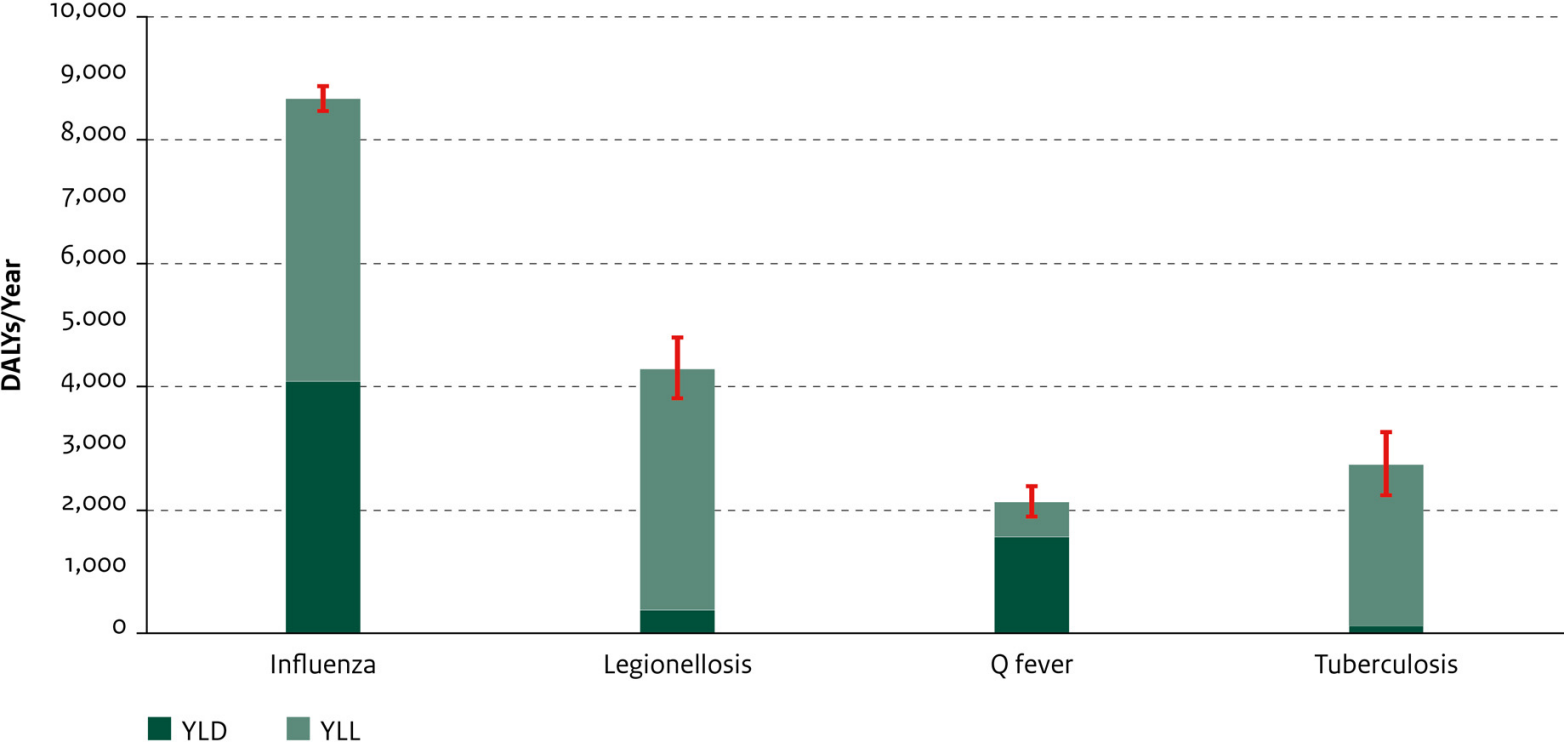
Average annual disease burden in the Netherlands in 2007–2011 for new cases of respiratory diseases. YLD and YLL components are shown separately. Red lines indicate 95% uncertainty intervals. (DALY = Disability-Adjusted Life Year, YLD = Years Lived with Disability, YLL = Years of Life Lost).

**Fig 8:**
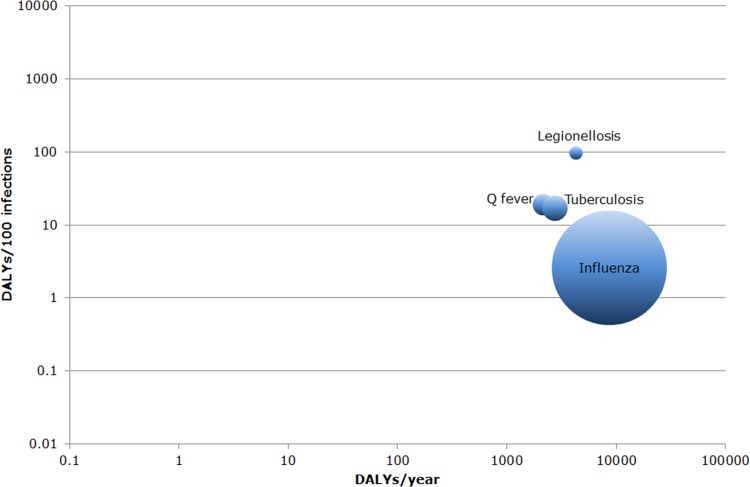
Ranking of respiratory diseases by disease burden at population/individual level in the Netherlands in 2007–2011. The area of each bubble is proportional to the average number of estimated annual cases. Both axes are on a logarithmic scale. (DALY = Disability-Adjusted Life Years, population level = number of DALYs/year, individual level = number of DALYs/100 infections).

Among the respiratory diseases, the greatest disease burden was estimated for influenza (8670 DALYs/year; 95% UI: 8468–8874) and legionellosis (4283 DALYs/year; 95% UI: 3819–4805) (Fig 7). This was due to high mortality for both diseases (432 estimated deaths (4580 YLL/year; 95% UI: 4474–4687) and 176 estimated deaths (3892 YLL/year; 95% UI: 3447–4389) per year, respectively). For influenza, a large share of the total disease burden (23%) was borne by persons aged 75 years or older, whereas for legionellosis 8% of the burden was carried by this age-group (see Figure S and Figure T in S2 Appendix). For all respiratory diseases, the disease burden at the population level was considerably larger than that at the individual level (Fig 8); the average individual-level disease burden for influenza in particular was relatively small (2.6 DALYs/100 infections).

## Discussion

This study represents the first estimation of the disease burden of a comprehensive set of 32 infectious diseases in the Netherlands using the pathogen- and incidence-based DALY approach. For food-related diseases, there is a long history of disease burden estimation [12], and a diverse selection of infectious diseases had been included in a previous comprehensive Dutch burden of disease study that used a different methodology [24, 25]. We found the estimated disease burden to vary greatly across a set of 32 pathogens possessing very different patterns of incidence and associated health outcomes. At the population level, invasive pneumococcal disease had the highest average annual burden, with an estimated 9444 DALYs/year, followed by influenza, at 8670 DALYs/year. At the individual level, rabies and variant Creutzfeldt-Jakob disease had the highest average disease burden, with 5081 and 3581 DALYs/100 infections respectively. However, the latter two diseases, together with diphtheria and poliomyelitis, occurred with a very low incidence. The current approach to disease burden estimation may be useful for many other diseases (e.g., human papilloma virus infection, infection with *Helicobacter pylori*, Lyme disease [26], psittacosis).

There are few previous studies that have published national or regional disease burden estimates focussing on infectious diseases [27, 28]. Mostly, national burden of disease studies include chronic diseases and injuries as well [29–37]. Most of these studies were conducted outside of Europe, do not use a pathogen-based approach, include a limited number of infectious diseases, and rely heavily on the GBD methodology and/or estimates of the World Health Organization (WHO). Differences in methodology or summary measures reported can complicate comparisons. For instance, the ambitious GBD 2010 project relies on complex statistical modelling to derive national estimates [21] for a wide range of infectious and non-communicable diseases. Although the GBD 2010 methods are transparent and suitably documented, the mortality and burden estimates were derived using (publicly-available) statistical tools that require an advanced level of modelling expertise, with the consequence that estimates are not easily reproducible without use of these tools. In our framework, it is relatively straightforward to regularly update burden estimates (e.g., annually), to construct and explore the impact of hypothetical scenarios such as the introduction of screening or improved vaccination programmes, and to identify the deficiencies of and directions for improving or enhancing existing surveillance systems.

### Sexually-transmitted infections

For most sexually-transmitted infections, the estimated disease burden was relatively high, which is attributable to either a severe natural history (e.g., HIV, hepatitis B, and hepatitis C infection), or a high incidence (e.g., chlamydia and gonorrhoea). Because most of the diseases in this group are not notifiable, estimating incidence was challenging. Furthermore, most surveillance systems focus on specific high-risk groups visiting clinics for sexually-transmitted infections. Therefore, estimating the degree of under-ascertainment for these diseases was also very difficult.

The present estimates only reflect the disease burden of new cases that occurred in the period 2007–2011. This means that, for diseases with chronic manifestations (e.g., hepatitis B, hepatitis C, and HIV infection), our estimation method did not take into account chronic cases infected prior to this period. The relatively low disease burden of hepatitis B infection in the Netherlands is likely due to low endemicity and the vaccination of high-risk groups. This targeted vaccination programme, started in 2002, has been shown to reduce the incidence of acute hepatitis B infection, chiefly by preventing hepatitis B infections in MSM [38]. Additionally, children at high risk (i.e., children with at least one parent born in a hepatitis B endemic country, and children whose mother tested positive for hepatitis B infection) have been vaccinated against hepatitis B within the National Immunisation Programme (NIP) since 2003. Universal hepatitis B vaccination was introduced to the Dutch NIP in 2011, i.e. much later than for other vaccine preventable diseases; this is expected to affect future disease burden estimates. An explanation for the relatively high YLL estimated for hepatitis B as compared with hepatitis C infection is the difference in the age distributions of notified cases: hepatitis C cases (mainly sexually transmitted cases among MSM) tend to be somewhat older and therefore have a lower risk of progressing to severe sequelae before the end of their natural lifetime.

The disease burden of HIV infection was based on newly registered HIV cases only, and using a natural history model that only partially reflects the impact of HAART; for instance, the disability weights for health outcomes following HIV infection were mostly derived before or shortly after introduction of HAART, and elicitation of up-to-date weights is required. So, modifying the HIV disease model to better take into account effects of HAART is clearly desirable. For hepatitis C infection, treatment options have recently changed, or will change in the near future, which will lead to improved prognosis and a subsequent reduction in disease burden which will also require modified parameters.

The relatively high disease burden of chlamydia, mainly driven by infertility among women, is striking. Chlamydia was the most frequently diagnosed bacterial sexually-transmitted infection in 2012, and the positivity rate has increased in recent years, especially in the younger age groups [39]. Despite the high disease burden, the Chlamydia Screening Implementation (CSI), a large scale trial offering annual screening to more than 300,000 young people in Amsterdam, Rotterdam and South-Limburg showed that population based chlamydia screening in the Netherlands is unlikely to be cost effective [40–42].

### Vaccine-preventable diseases

The estimated disease burden for most of the vaccine-preventable diseases is relatively low, testimony to the effectiveness of the NIP [43–45] which was begun in the 1950s and has achieved a high coverage [46]. It is vital to maintain this attained level of coverage in the future to prevent resurgence of those vaccine-preventable diseases that are currently under control. The current disease burden estimates for vaccine-preventable diseases are consistent with the general observation that pertussis is not yet under control [47], and that current vaccines against invasive bacterial disease (*H*. *influenzae*, meningococcal, and in particular pneumococcal infection) only protect against certain serotypes.

For pertussis, 2012 (the year subsequent to our study period) was an epidemic year, and the estimated disease burden in 2012 was more than twice as high (6842 DALYs and 63 deaths) than the estimated mean annual disease burden in the period 2007–2011 (3235 DALYs/year and 29 deaths). Although the number of officially reported pertussis deaths (2 in the period 2007–2011 [48]) might be under-estimated to some extent, especially among older people, an annual average of 29 pertussis deaths during 2007–2011 and 63 deaths during 2012 is probably unrealistic for the Dutch context. In comparison: in England 18 of 46 estimated pertussis deaths were officially reported and 9 annual pertussis deaths were estimated in total [49]. Because the case fatality rates in the disease model are not stratified by age-group, it is likely that the disease burden of pertussis is over-estimated.

Current vaccines do not cover all *H*. *influenzae*, meningococcal and pneumococcal serotypes; however, our results illustrate the effectiveness of the NIP because the vaccine-preventable serotypes are under control. Namely, in the period 2007–2011, only 30% of the disease burden of invasive *H*. *influenzae* disease was caused by serotype b (Hib), only 4% of the disease burden of invasive meningococcal disease was due to Men C (86% was caused by Men B, for which vaccination is not included in the NIP), and 25% of the disease burden of invasive pneumococcal disease was caused by a serotype covered by the 7-valent pneumococcal vaccine (PCV7) that was used until 2011, before being replaced by PCV10. For invasive pneumococcal disease, for which vaccination was introduced in the NIP in 2006, our national data indicated that this proportion decreased from 40% in 2007 to 15% in 2011. However, although pneumococcal conjugate vaccination decreased the occurrence of vaccine-type invasive pneumococcal disease, non-vaccine type invasive pneumococcal disease increased due to serotype replacement, thereby reducing the overall benefit of vaccination [50].

The actual disease burden for pneumococcal disease is even higher than presented, because we computed the disease burden for the *invasive* form of pneumococcal disease only; the disease burden of otitis media and non-invasive pneumonia was not included. Non-invasive forms of *H*. *influenzae* infection were also excluded, meaning that comparison of the disease burden with other diseases should take this restriction to invasive forms into account. Mortality, and thus disease burden, associated with invasive pneumococcal disease might have been slightly over-estimated, because Dutch data indicates a mortality risk of 12% [50] which results in 7582 DALY per year, whereas the mortality risk in the disease model was set to 10–20%.

The annual disease burden for vaccine-preventable diseases can fluctuate enormously due to outbreaks that occur mainly among unvaccinated members of orthodox religious communities [51–54]. The estimated disease burden due to the measles epidemic in 2013 (9319 DALYs) was 139 times higher than the mean annual disease burden in the inter-epidemic period 2001–2012 (67 DALYs/year). The total estimated disease burden for the poliomyelitis outbreak in 1992/1993 was 442 DALYs (71 reported cases), whereas there were no cases and thus no disease burden in the period 2007–2011. The rubella outbreak in 2004/2005 had a total estimated disease burden of 8449 DALYs (415 reported cases), compared with <1 DALY/year in the period 2007–2011; 99.7% of this disease burden could be attributed to CRS. Prevention of CRS was the principal motivation for introducing rubella vaccination. For mumps, the estimated disease burden was quite low despite the relatively high number of reported cases in the period 2007–2011. Even in the epidemic year 2011, the estimated disease burden for mumps remained low (6 DALYs), due to the fact that the risk of severe disease and mortality is relatively low.

### Food-related diseases

Of the food-related diseases, the highest average annual disease burden was estimated for toxoplasmosis, campylobacteriosis, norovirus infection, and salmonellosis (3593, 3314, 1647, and 1375 DALYs/year, respectively). The high population-level burden of the aforementioned four diseases is mainly driven by the large number of persons infected (and relatively high severity of toxoplasmosis). The disease burden estimates, although based on the average incidence over a five-year period, are comparable to previously published estimates for the year 2009 only: 3620, 3250, 1480, and 1270 DALYs for toxoplasmosis, campylobacteriosis, norovirus infection, and salmonellosis respectively [12]. The mean estimated individual-level burden for food-related diseases other than variant Creutzfeldt-Jakob disease, toxoplasmosis, and listeriosis is very low (≤17 DALYs/100 infections).

In the disease burden estimation approach developed by Havelaar *et al*. [12] (which we applied to all food-related diseases except variant Creutzfeldt-Jakob disease and shigellosis), transition probabilities for the severity of health outcomes were integral to the burden calculation for several food-related diseases; for others, separate incidence estimates were made for cases in the general population, cases visiting their general practitioner, and hospitalised patients. The latter method makes use of available national cohort studies for a number of health outcomes (incidence derived from population-wide studies, general practitioner visits, hospital admissions), which are attributed to different pathogens through laboratory examination of faecal specimens. The two approaches–outcome trees with transition probabilities specified between health outcomes versus the direct use of incidence data for each health outcome–are equivalent if the transition probabilities are derived from the same national data sources.

### Respiratory diseases

The estimated disease burden for most of the respiratory diseases is relatively high, reflecting simultaneously the large impact of mortality and the large number of incident cases (e.g., influenza). As for many vaccine-preventable diseases, incidence and thus the disease burden of influenza and Q fever can fluctuate enormously per year. The burden of Q fever in 2009 (peak year of cases due to an outbreak that started in 2007 [55]) was estimated at 6162 DALYs compared with 2143 DALYs/year in the period 2007–2011. The estimated disease burden of influenza for 2009 (the year of the H1N1 pandemic) was almost twice as high (16,378 DALYs) as the estimated average annual disease burden for the period 2007–2011 (8670 DALYs/year). Note that the number of incident cases estimated for the 2009 calendar year includes both the unusually high peak observed for the 2008/2009 influenza season and the early peak of the 2009/2010 H1N1 pandemic; as a consequence the annual average incidence of influenza in the period 2007–2011 is unduly influenced by the incidence in 2009.

The estimated disease burden for legionellosis is considerable; this could be due to several factors. Firstly, the large legionellosis burden may be attributable to over-estimated incidence, due to the relatively high multiplication factor derived by relying on a German study on community-acquired and hospitalised pneumonia patients [56] to inform certain disease model parameters. The proportion of legionellosis among pneumonia cases reported in the literature can vary substantially [56–58], and because pneumonia occurs frequently in the population, the proportion assumed can have a significant effect on the estimated incidence of legionellosis. Furthermore, legionellosis often has a more severe course than other respiratory diseases [59] (e.g., Q fever), and is therefore more likely to be notified.

Second, the Dutch surveillance system is considered to be of high quality [60, 61]. The number of reported cases of legionellosis in the Netherlands is relatively high compared with other countries [62], suggesting that under-reporting should be relatively low, partly because routine use of the *Legionella pneumophila* urinary antigen test has become standard of care in patients with severe community-acquired pneumonia in many Dutch hospitals [63].

Third, in the period 2007–2011, 40% of the reported legionellosis cases (range 32% in 2010 to 46% in 2007) were travel-related and thus were likely to have been acquired abroad and cannot be prevented through the implementation of national control measures. The increase of legionellosis in 2010, the year with the lowest proportion (32%) of travel-related legionellosis cases in 2007–2011, may have been related to weather conditions (i.e., the unusually hot summer of 2010, which was followed by extensive rainfall) or to other environmental factors [62, 64, 65]. This exceptional year 2010 had a marked effect on the annual disease burden estimate; the burden in 2010 was 5863 DALYs compared with an average of 4283 DALYs/year for the total period 2007–2011.

There are several limitations to the estimated disease burden of tuberculosis, as specifying the disease progression pathway is not straightforward. Firstly, migration patterns have considerable influence on tuberculosis incidence. In recent years, the proportion of patients with extra-pulmonary tuberculosis (which can differ in clinical severity from pulmonary tuberculosis) has increased [66], and is notably higher than in other European countries [67]. This is due to an increased number of imported cases among asylum seekers originating from Somalia [66]. Such recent changes in clinical manifestations are not captured by the disability weights used in the current tuberculosis disease model. Secondly, we note a risk of double counting of active tuberculosis cases. The number of active tuberculosis cases that develop from latent infection is determined by the disease model, by first back-calculating the total number of infections from the number of reported cases. However, reported cases actually represent a mixture of active tuberculosis cases following both primary and latent infection, and therefore some active tuberculosis cases following latent infection may effectively be ‘counted twice’. Finally, the transition probability by which patients progress to active tuberculosis following primary infection (specified as the range 5–10%) is expected to be lower for the Netherlands compared with other countries due to the practice of screening and preventive treatment of latently infected tuberculosis contacts and of other high risk groups. Through preventive treatment, the risk of developing active tuberculosis can be reduced by 60–90% [68].

### Limitations

In addition to the general methodological issues in computing DALYs based on an EU harmonised methodology for infectious diseases addressed above, there are a number of limitations to the present study that should be considered when interpreting the findings. First, disease model parameters were specified in collaboration with European experts to ensure the plausibility of the estimated disease burden. This may have introduced bias, because diseases for which preliminary disease burden calculations were high received more attention and provoked more discussion regarding the correctness of model parameters compared with diseases with a low estimated disease burden.

Second, most parameters (i.e., case-fatality rates, transition probabilities of progressing to severe sequelae) were derived from studies among reported cases, and so applying the same parameters also to non-reported cases may not always be correct. Although age-group and sex-specific values for case-fatality rates and transition probabilities were specified if published or otherwise available, for most diseases only age-independent values were located. This places a significant limitation on burden computation when progression to a severe sequela or to death is dependent on age, as already noted above for pertussis.

Third, for almost all of the diseases investigated, adjustment for under-ascertainment/reporting of notified cases was carried out via age- and sex-independent multiplication factors, because there were insufficient data to specify stratified multiplication factors. As a consequence, sex- and/or age-groups with relatively more notified severe cases may be over-represented, and groups with fewer notified severe cases may be under-represented [9]. Such bias would have greater consequences for those diseases with long natural histories.

Fourth, co-morbidity with chronic disease or co-infection with other pathogens was not considered. Various methods for adjusting disability weights to capture the severity of simultaneous health outcomes, and for cause-specific YLL attribution in the case of fatal comorbidity have been explored, but have not yet reached a satisfactory level of development to permit straightforward incorporation in the current methodology.

Variability in annual incidence over time was not incorporated, since we calculated the mean incidence and burden over the period 2007–2011. Averaging incidence across years does not affect the uncertainty regarding the number of incident cases–and hence the disease burden–for an ‘average’ year; however, it does conceal potentially interesting variation, such as outbreaks. For several diseases with periodic variation in incidence (e.g., measles, pertussis), we have discussed the considerable differences in estimated disease burden between outbreak years and other years.

Finally, the present national disease burden estimates were derived under the ‘steady-state’ assumption; i.e., both the transmission and pathogenicity of infections and the size and age-structure of the susceptible population are considered static. Demographic change, due to population ageing and changing migration patterns, diminishing or increasing natural immunity to certain infectious agents, and new interventions would be expected to influence the projected future disease burden of most, if not all, pathogens [69, 70]. Therefore, caution must be taken when extrapolating the estimated disease burden to future years.

As a basis both for future extension and enhancement within the Dutch setting, and for application to other countries, the present study–because of its transparency in methods and data acquisition/adjustment–is eminently suitable. At the national level, our estimates can serve as a ‘baseline’ snapshot of the infectious disease burden to population health; such a baseline will be useful for evaluation of the impact of future public health initiatives. Future plans include establishing the routine annual calculation of disease burden for the current set of infectious diseases, improving the estimation of multiplication factors, investigation and visualisation of temporal trends, improvement of currently used outcome trees, and the incorporation of additional disease models into the existing set (e.g., human papilloma virus infection, infection with *Helicobacter pylori*, Lyme disease, psittacosis).

## Conclusions

The current results represent a first attempt to assess the disease burden of a comprehensive set of infectious diseases in the Netherlands. Disease burden methodology provides a new perspective on infectious disease surveillance data; it avoids the devotion of excessive attention to rare infections with dramatic outcomes and the neglect of common disorders. In general, the disease burden also reflects the balance between the threat posed by an infection and the effectiveness of prevention against this infection. Our study highlighted the high disease burden attributable to invasive pneumococcal disease and influenza, and the uneven apportioning of burden for these diseases across age-groups. A low estimated burden for those diseases included in the NIP stresses the need for the continued support of these strategies, whereas a high disease burden for diseases covered by the NIP suggests that additional preventive measures may be needed. For prioritising interventions and preventive measures, estimates of trends in disease burden are undoubtedly informative and may reflect the overall impact of control efforts. Together with other factors such as the availability of preventive strategies, societal costs, and public perception, disease burden estimates provide vital contributions to defining and supporting public health policy. The experience accumulated over the course of this project in defining strategies for data compilation and adjustment (a process subject to continual improvement), the practicalities of disease model parameterisation and disease burden computation, and interpretation of the findings can usefully serve to support other countries when designing their own national disease burden estimation exercises [71].

## Supporting Information

S1 Appendix Derivation of multiplication factors. (DOCX)

S2 Appendix Sex and age-group specific disease burden estimates. (DOCX)
