# Supplementary material for: Disease Burden of 32 Infectious Diseases in the Netherlands, 2007-2011
Source: PLoS One. 2016 Apr 20;11(4):e0153106. doi: 10.1371/journal.pone.0153106 (PMC4838234; doi:10.1371/journal.pone.0153106)
Supplement: S1 Appendix — (DOCX) [file pone.0153106.s001.docx]

**S1 Appendix: Derivation of multiplication factors**

Physicians, laboratories and heads of health-care institutions are required to report 42 notifiable infectious diseases to the regional Public Health Services [[1](#_ENREF_1)]. The regional Public Health Services then report these notifications to the national registration system, OSIRIS, an important source of information on the occurrence of infectious diseases in the Netherlands. However, for a number of diseases there is under-ascertainment, under-reporting and delay in reporting [[2](#_ENREF_2)]. Because of this under-estimation, it is important to adjust the reported case numbers using multiplication factors (Table S1) before computing disease burden. For detailed information on disease-specific surveillance systems and data sources, see State of Infectious Diseases in the Netherlands, 2013 [[3](#_ENREF_3)].

**Table S1.** Multiplication factors chosen for all 32 diseases, and rationale for choice.

| **Disease** | **MF(s) chosen** | **Explanation** |
| --- | --- | --- |
|  |  |  |
| **Sexually-transmitted infections** | | |
| Chlamydia | UA: 1 UR: 1.111 | We assumed 10% under-reporting to account for people who are tested in other settings (gynaecologist, directly at laboratory, self-test). |
| Gonorrhoea | UE: 2.53 | MF is based on absolute numbers from SOAP and LINH. |
| Hepatitis B infection | UA: 1.33  UR: Uniform(1.20,1.22) | For under-ascertainment, we assume that 75% of all symptomatic cases is reported; this is based on England and Wales data from 1992-1996 [[4](#_ENREF_4)]. For under-reporting, the lower bound was derived from a 1996 audit, and the upper bound was taken from Swaan *et al*.'s (unpub.) study of reporting completeness (weighted mean of 83.1%) by 13 South Holland laboratories, 2005-2010. |
| Hepatitis C infection | UE: Uniform(1, 5.12)*29/30 + Pert(0, 47, 464.4)*1/30 | MFs were calculated for MSM only; it was assumed that there is no under-estimation for non-MSM risk groups. MF is a weighted sum derived from the estimated incidences of HCV among HIV-positive and HIV-negative MSM, weighted for the proportion of notified cases represented by the two respective groups. Note that the estimated annual incidence is quite uncertain (95% CI: 855-1662) due to the wide MF distribution specified for HIV-negative MSM, itself attributable to the wide uncertainty range in the incidence rate estimated for this group. This MF was only applied to males aged 20-69 years; for all other age groups and females, MF was set to 1. |
| HIV infection | UE: 1 | No MF is available to estimate actual incidence from diagnoses. Because it takes about two years for the HIV diagnosis register to become complete, we corrected for reporting delay (to estimate completeness as of 2012, the number of diagnoses in 2011 was multiplied by 11% and the number of diagnoses in 2010 was multiplied by 3%). |
| Syphilis | UE: 4.21 | MF based on absolute numbers from SOAP and LINH. |

| **Vaccine-preventable diseases** | | |
| --- | --- | --- |
| Diphtheria | - | - |
| Invasive *H.  influenzae* infection | UE: Uniform(1.05,1.20) | Approximately 83-95% of isolates are sent to the Netherlands Reference Laboratory for Bacterial Meningitis, implying an MF of 1.05 to 1.20. |
| Invasive  meningococcal disease | UE: 1.05 | Meningococcal disease is a notifiable disease and notifications are cross-checked with data of the Netherlands Reference Laboratory for Bacterial Meningitis. Therefore coverage will be very high, around 95%, implying an MF of 1.05. |
| Invasive  pneumococcal disease | UE: Uniform(1.05,1.20) | Approximately 83-95% of isolates are sent to the Netherlands Reference Laboratory for Bacterial Meningitis, implying an MF of 1.05 to 1.20. |
| Measles | UE: Uniform(11.11,14.93) | According to Van Isterdael *et al*. (2004) [[5](#_ENREF_5)] 9% of all measles cases were reported (MF=11.11). Wallinga *et al*. (2003) [[6](#_ENREF_6)] estimated that 6.7% of all infections were reported (MF=14.93). Both estimates are based on the 1999/2000 measles outbreak in the Netherlands. |
| Mumps | UA: 1.84  UR: 1 | Under-ascertainment MF is based on Greenland *et al*. (2010) [[7](#_ENREF_7)]. No data on under-reporting were available; we applied MF=1. |
| Pertussis | UE: 21.9 (0-9 yrs);   25 (>9 yrs) | MFs are based on Pienter-2 serosurvey data [[8](#_ENREF_8)], corrected for the proportion symptomatic, separately for children 0-9 years and for persons >9 years. |
| Poliomyelitis | UE: 1 | Because there were 0 cases in the period 2007-2011, this MF was only applied to calculate the disease burden of the poliomyelitis outbreak in 1992/1993. Because of the severity of the disease, we assumed that all cases are identified. |
| Rabies | UE: 1 | Because of disease severity, we assumed that all cases are identified. |
| Rubella | UE: Uniform(11.11,14.93) | No information available. We therefore used the MF for measles as a proxy (the clinical picture is probably less clear compared with measles, except for congenital rubella syndrome). |
| Tetanus | UE: Uniform(1.0,1.41) | Range of 1 to 1.41 was based on expert opinion that the MF would be close to 1.0 (set as lower bound), and a Danish study suggesting 1.41 (upper bound) [[9](#_ENREF_9)]. |
| **Food-related diseases** | | |
| Campylobacteriosis,  Cryptosporidiosis,  Giardiasis,  Hepatitis A infection,  Listeriosis,  Norovirus infection,  Salmonellosis,  Toxoplasmosis,  Infection with STEC O157 |  | For these food-related diseases, an estimation method developed by Havelaar *et al*. was used that is specific for the Dutch context [[10](#_ENREF_10), [11](#_ENREF_11)]. |
| Shigellosis | UE: PERT(1.2,11.6,49.6) | As a proxy, an MF calculated for salmonellosis (based on the proportion of (a) the number of estimated salmonellosis cases in 2009 [[10](#_ENREF_10)] and (b) the mean number of reported salmonellosis cases in 2007-2011) was used. |
| vCreutzfeldt-Jakob disease | UE: 1 | No correction due to the 100% case-fatality rate. Cases may be missed, especially elderly patients, if symptoms are attributed to a different cause. |

| **Respiratory diseases** | | |
| --- | --- | --- |
| Influenza | UA: Uniform(4.12,5.13)  UR: 1 | MF for under-ascertainment is based on the estimated proportion of people who go to the general practitioner when they have ILI symptoms (source: “Grote Griepmeting” [[12](#_ENREF_12)]). MF ranged from 4.12 to 5.13 in the period 2007-2011 (2007: 4.43, 2008: 4.12, 2009: 4.19, 2010: 5.13 and 2011: 4.42). |
| Legionellosis | UA: 1  UR: PERT(9.95,11.03,24.14) | UR is based on Dutch notification data, the estimated number of pneumonia cases in the Netherlands [[13](#_ENREF_13), [14](#_ENREF_14)], and the expected proportion with diagnosis legionellosis [[15-17](#_ENREF_15)]. |
| Q fever | UE: PERT(0.75,1.575,3.25)  (0-14 yrs)  PERT(2.4,5.04,10.4)  (15+ yrs) | Van der Hoek *et al*. (2012) [[18](#_ENREF_18)] showed that in the highest incidence areas of the Netherlands in 2009, one notification represented 12.6 infections (95% CI: 6-26) (either symptomatic or asymptomatic). In the international literature, a symptomatic percentage of 12.5% (0-14 years) and 40% (15 years or older) is applied [[19-21](#_ENREF_19)]. Therefore, we estimate that one notification represents 1.6 (0-14 years) or 5 symptomatic cases (15+ years). |
| Tuberculosis | UA: 1  UR: Uniform(1.08,1.16) | UR is based on Van Hest *et al*. (2007) [[22](#_ENREF_22)]: 1.08 (record-linkage study) to 1.16 (capture-recapture analysis). |

UE = under-estimation, UA = under-ascertainment, UR = under-reporting. SOAP= web-based surveillance system for sexually-transmitted infections, LINH = National Information Network Primary Care, MSM=men who have sex with men.

**References**

1. van Vliet H. Geschiedenis van meldingsplicht. [History of notification]. Tijdschrift voor infectieziekten. 2009;4(2):51-60. Dutch.

2. de Melker HE, Conyn-van Spaendonck MAE, Sprenger MJ. Infectieziekten in Nederland: epidemiologie, diagnostiek en bestrijding. [Infectious diseases in the Netherlands: epidemiology, diagnostics and control]. The Hague: National Institute for Public Health and the Environment (RIVM); 1997. Dutch.

3. van Lier A, McDonald S, Bouwknegt M, Bijkerk P, Havelaar A, Kretzschmar M, et al. Disease burden of infectious disease in the Netherlands. In: Bijkerk P, van Lier A, McDonald S, Kardamanidis K, Fanoy EB, Wallinga J, et al., editors. State of infectious diseases in the Netherlands, 2013. Bilthoven: National Institute for Public Health and the Environment (RIVM); 2014 (RIVM report 150205001). <http://www.rivm.nl/bibliotheek/rapporten/150205001.pdf>.

4. Ramsay M, Gay N, Balogun K, Collins M. Control of hepatitis B in the United Kingdom. Vaccine. 1998;16 Suppl:S52-5.

5. van Isterdael CE, van Essen GA, Kuyvenhoven MM, Hoes AW, Stalman WA, de Wit NJ. Measles incidence estimations based on the notification by general practitioners were suboptimal. J Clin Epidemiol. 2004;57(6):633-7.

6. Wallinga J, Teunis P, Kretzschmar M. Reconstruction of measles dynamics in a vaccinated population. Vaccine. 2003;21(19-20):2643-50.

7. Greenland K, Whelan J, Fanoy E, Borgert M, Hulshof K, Yap KB, et al. Mumps outbreak among vaccinated university students associated with a large party, the Netherlands, 2010. Vaccine. 2012;30(31):4676-80.

8. de Greeff SC, de Melker HE, van Gageldonk PG, Schellekens JF, van der Klis FR, Mollema L, et al. Seroprevalence of pertussis in The Netherlands: evidence for increased circulation of Bordetella pertussis. PLoS One. 2010;5(12):e14183.

9. Christiansen AH, Andersen PH. [Incidence of tetanus in Denmark, 1983-2000]. Ugeskr Laeger. 2005;167(7):757-9.

10. Havelaar AH, Haagsma JA, Mangen MJ, Kemmeren JM, Verhoef LP, Vijgen SM, et al. Disease burden of foodborne pathogens in the Netherlands, 2009. Int J Food Microbiol. 2012;156(3):231-8.

11. Haagsma JA, van der Zanden BP, Tariq L, van Pelt W, van Duynhoven YTPH, Havelaar AH. Disease burden and costs of selected foodborne pathogens in the Netherlands, 2006. Bilthoven: National Institute for Public Health and the Environment (RIVM); 2009 (RIVM report 330331001).

12. Friesema IH, Koppeschaar CE, Donker GA, Dijkstra F, van Noort SP, Smallenburg R, et al. Internet-based monitoring of influenza-like illness in the general population: experience of five influenza seasons in The Netherlands. Vaccine. 2009;27(45):6353-7.

13. Dijkstra F, van Gageldonk-Lafeber AB, Brandsema P, Friesema IHM, Robert-Du Ry van Beest Holle M, van der Lubben IM, et al. Jaarrapportage surveillance respiratoire infectieziekten 2007/2008. [Annual report surveillance respiratory infectious diseases 2007/2008]. Bilthoven: National Institute for Public Health and the Environment (RIVM); 2008 (RIVM-letter report 210231003). Dutch.

14. Brandsema PS, Dijkstra F, van Gageldonk-Lafeber AB, Snijders BEP, Meijer A, van der Hoek W. Jaarrapportage surveillance respiratoire infectieziekten 2010. [Annual report surveillance respiratory infectious diseases 2010]. Bilthoven: Rijksinstituut voor Volksgezondheid en Milieu (RIVM); 2011 (RIVM-letter report 210231008). Dutch.

15. von Baum H, Ewig S, Marre R, Suttorp N, Gonschior S, Welte T, et al. Community- acquired Legionella pneumonia: new insights from the German competence network for community acquired pneumonia. Clin Infect Dis. 2008;46(9):1356-64.

16. Schneeberger PM, Dorigo-Zetsma JW, van der Zee A, van Bon M, van Opstal JL. Diagnosis of atypical pathogens in patients hospitalized with community-acquired respiratory infection. Scand J Infect Dis. 2004;36(4):269-73.

17. Braun JJ, de Graaff CS, de Goey J, Zwinderman AH, Petit PL. Buiten het ziekenhuis opgelopen pneumonie: verwekkers en beloop bij patiënten opgenomen in een algemeen ziekenhuis. [Community-acquired pneumonia: pathogens and course in patients admitted to a general hospital]. Ned Tijdschr Geneeskd. 2004;148(17):836-40. Dutch.

18. van der Hoek W, Hogema BM, Dijkstra F, Rietveld A, Wijkmans CJ, Schneeberger PM, et al. Relation between Q fever notifications and Coxiella burnetii infections during the 2009 outbreak in The Netherlands. Euro Surveill. 2012;17(3):20058.

19. Dupuis G, Petite J, Peter O, Vouilloz M. An important outbreak of human Q fever in a Swiss Alpine valley. Int J Epidemiol. 1987;16(2):282-7.

20. Maurin M, Raoult D. Q fever. Clin Microbiol Rev. 1999;12(4):518-53.

21. Dijkstra F, van der Hoek W, Wijers N, Schimmer B, Rietveld A, Wijkmans CJ, et al. The 2007-2010 Q fever epidemic in The Netherlands: characteristics of notified acute Q fever patients and the association with dairy goat farming. FEMS Immunol Med Microbiol. 2012;64(1):3-12.

22. van Hest NA, Smit F, Baars HW, De Vries G, De Haas PE, Westenend PJ, et al. Completeness of notification of tuberculosis in The Netherlands: how reliable is record- linkage and capture-recapture analysis? Epidemiol Infect. 2007;135(6):1021-9.
