# Supplementary material for: Disease Burden of 32 Infectious Diseases in the Netherlands, 2007-2011
Source: PLoS One. 2016 Apr 20;11(4):e0153106. doi: 10.1371/journal.pone.0153106 (PMC4838234; doi:10.1371/journal.pone.0153106)

**S2 Appendix: Age-group specific disease burden estimates**

In this Appendix, we graphically present disease burden estimates stratified by sex and five-year age-group (in DALYs/year, split into YLD and YLL) for 22 of the infectious diseases investigated (those diseases for which disease burden was estimated using the BCoDE toolkit).

**Fig A.** **Sex- and age-group specific disease burden of chlamydia, from the BCoDE toolkit.**


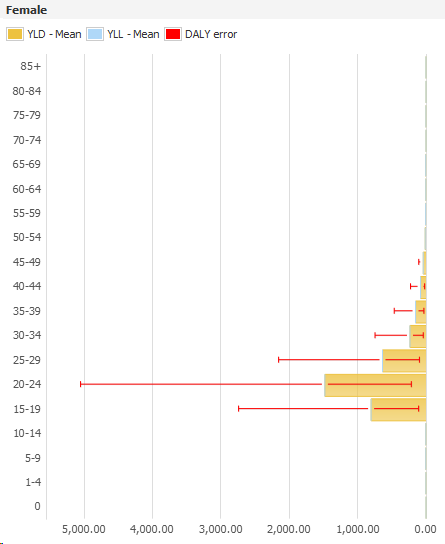

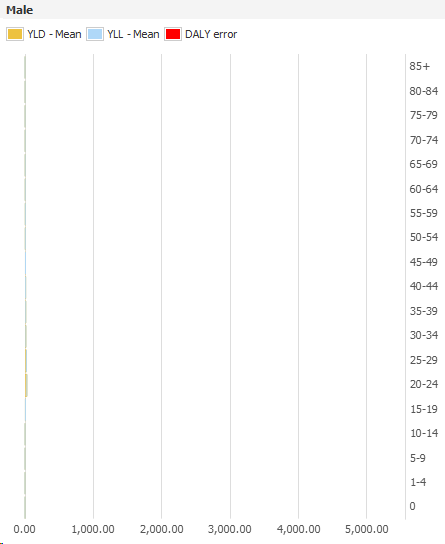


**Fig B. Sex- and age-group specific disease burden of gonorrhoea, from the BCoDE toolkit.**


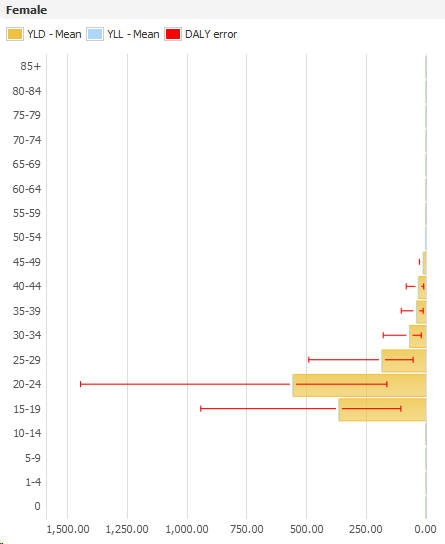

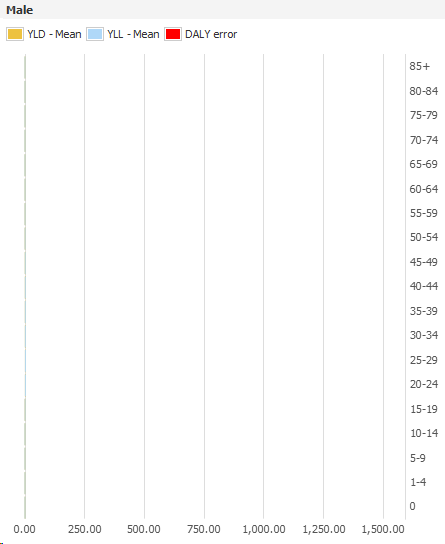


**Fig C. Sex- and age-group specific disease burden of hepatitis B infection, from the BCoDE toolkit.**


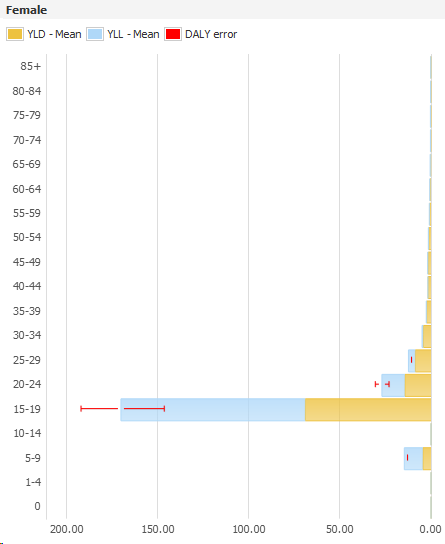

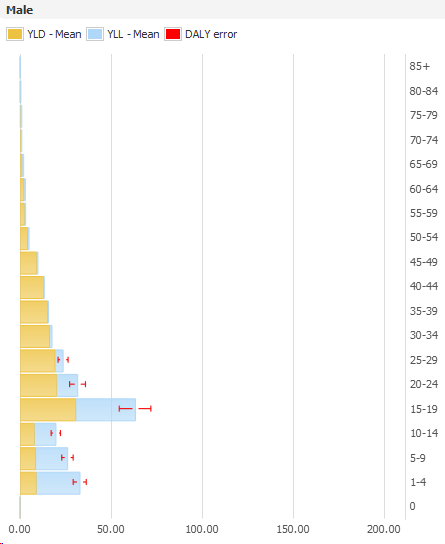


**Fig D. Sex- and age-group specific disease burden of hepatitis C infection, from the BCoDE toolkit.**


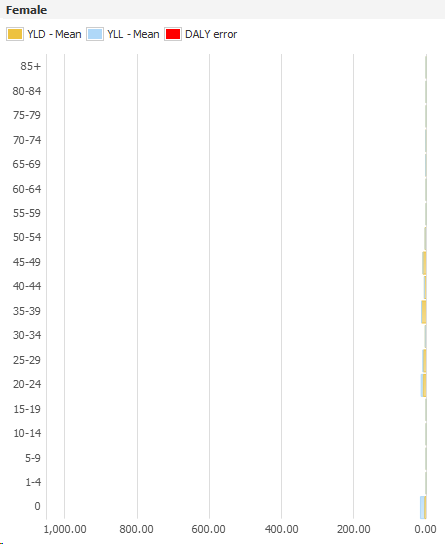

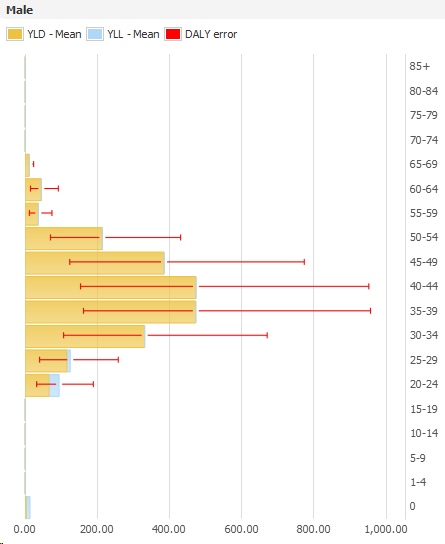


**Fig E. Sex- and age-group specific disease burden of HIV infection, from the BCoDE toolkit.**


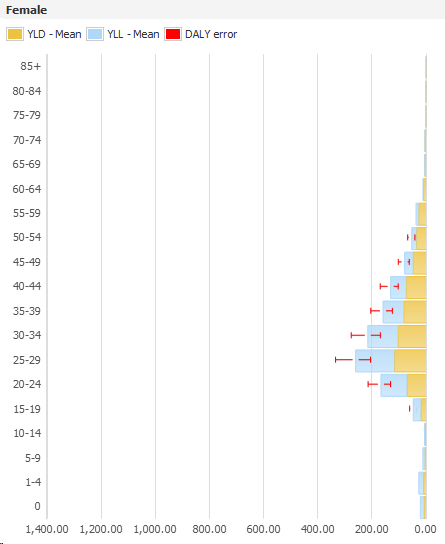

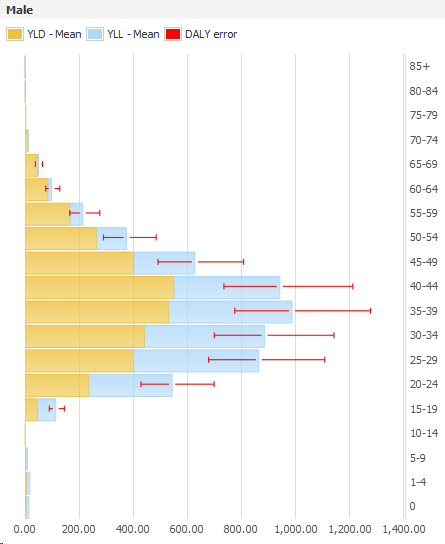


**Fig F. Sex- and age-group specific disease burden of syphilis, from the BCoDE toolkit.**


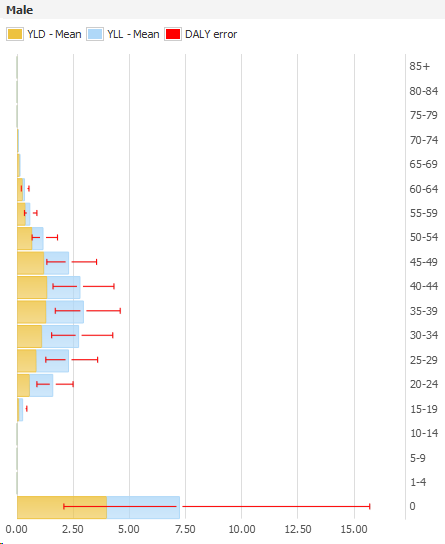

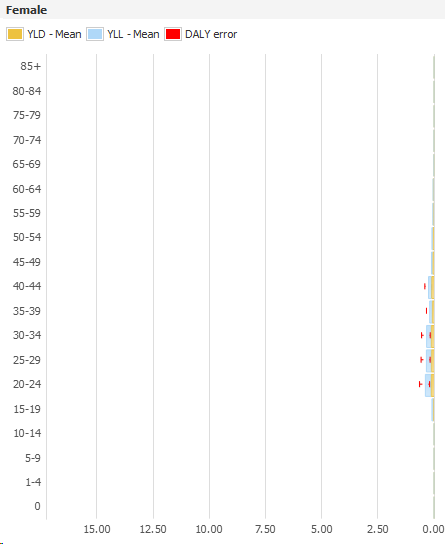


**Fig G. Sex- and age-group specific disease burden of invasive *H. influenzae* infection, from the BCoDE toolkit.**


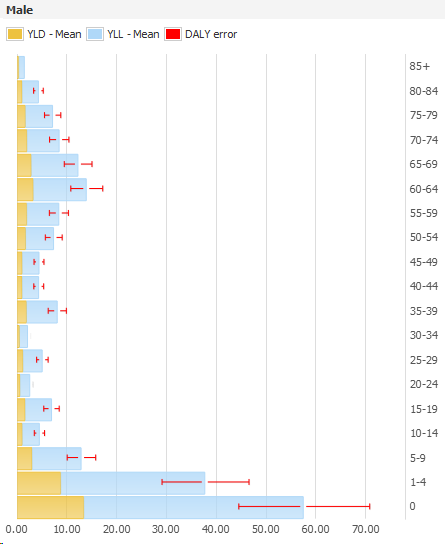

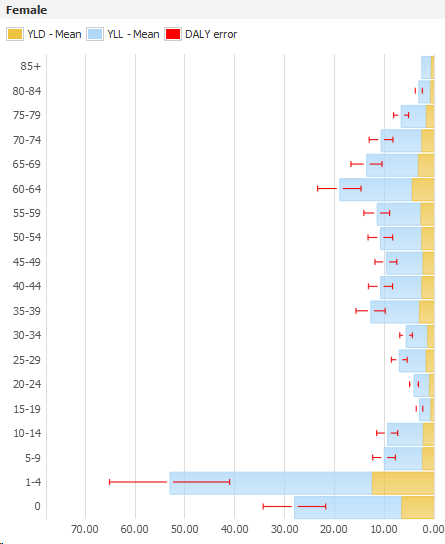


**Fig H. Sex- and age-group specific disease burden of invasive meningococcal disease, from the BCoDE toolkit.**


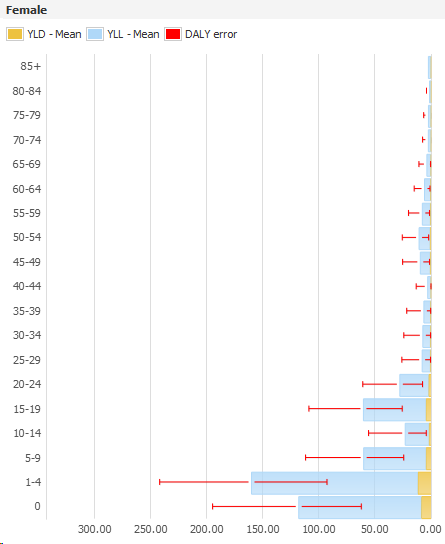

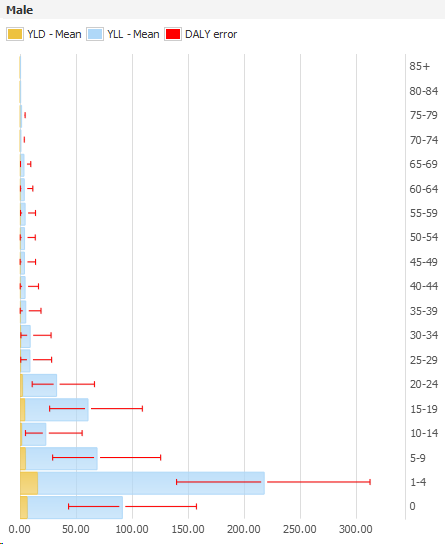


**Fig I. Sex- and age-group specific disease burden of invasive pneumcoccal disease, from the BCoDE toolkit.**


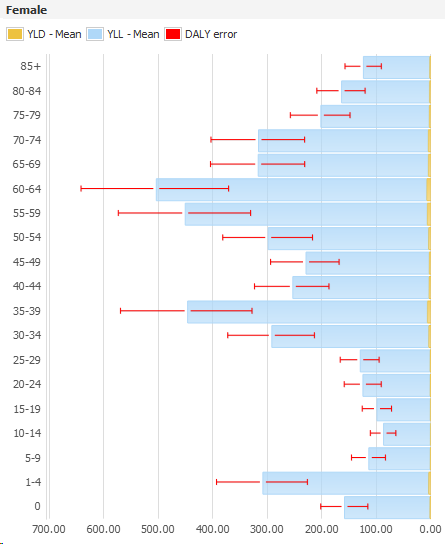

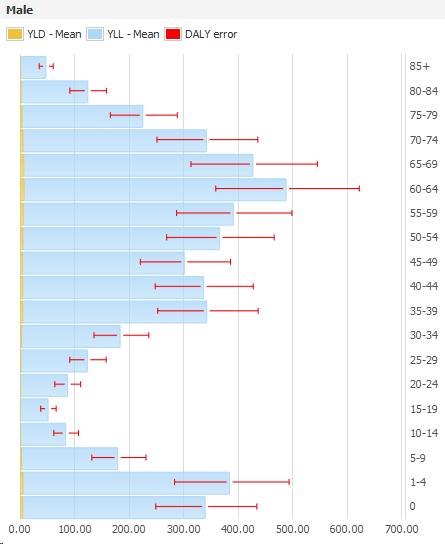


**Fig J. Sex- and age-group specific disease burden of measles, from the BCoDE toolkit.**


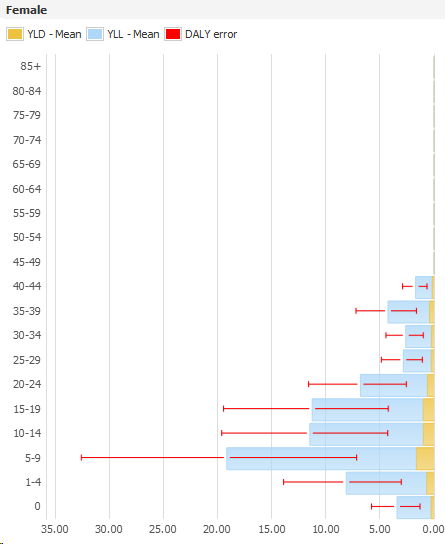

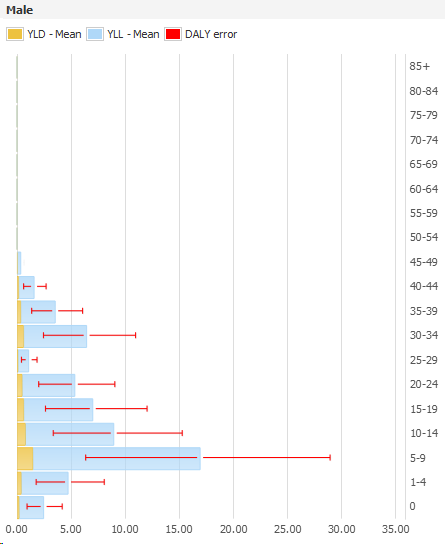


**Fig K. Sex- and age-group specific disease burden of mumps, from the BCoDE toolkit.**


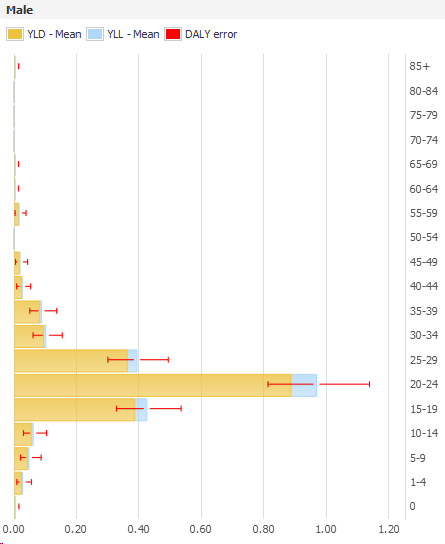

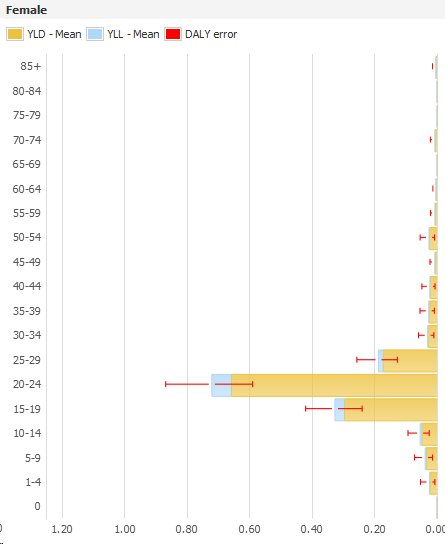


**Fig L. Sex- and age-group specific disease burden of pertussis, from the BCoDE toolkit.**


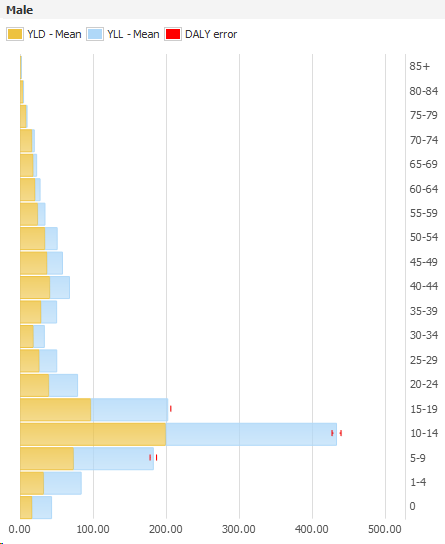

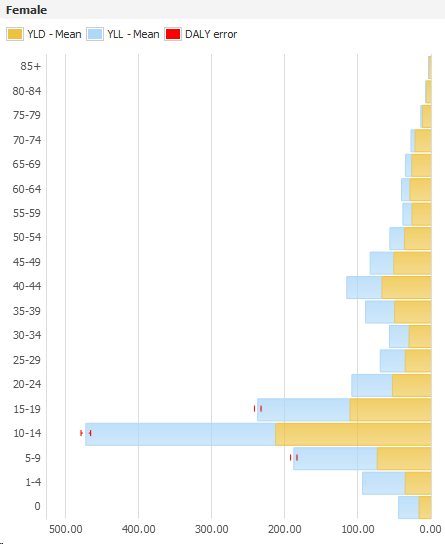


**Fig M. Sex- and age-group specific disease burden of rabies, from the BCoDE toolkit.**


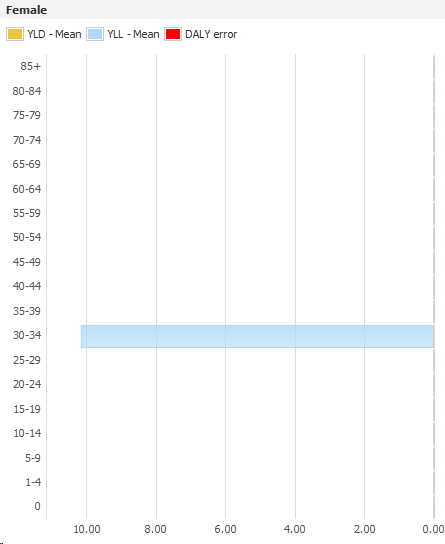

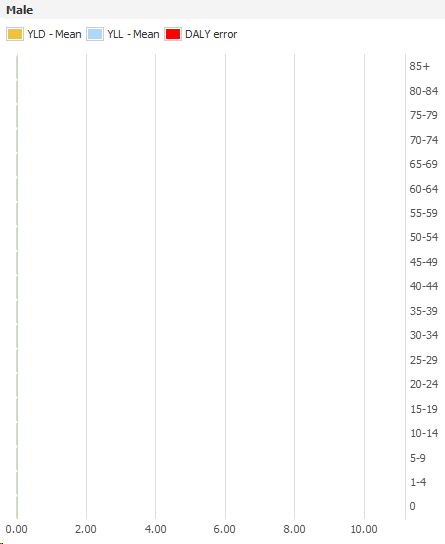


**Fig N. Sex- and age-group specific disease burden of rubella, from the BCoDE toolkit.**


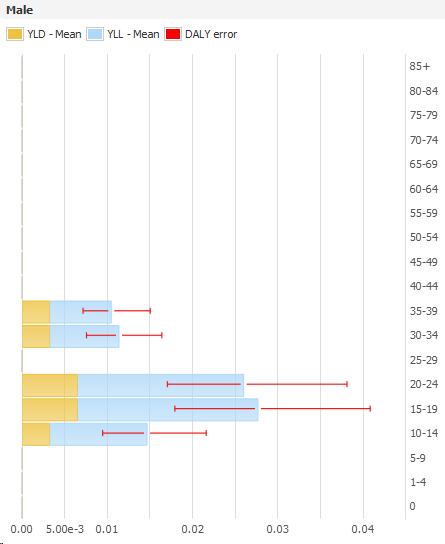

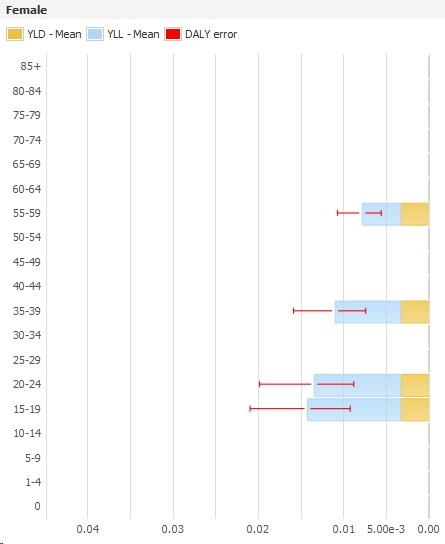


**Fig O. Sex- and age-group specific disease burden of tetanus, from the BCoDE toolkit.**


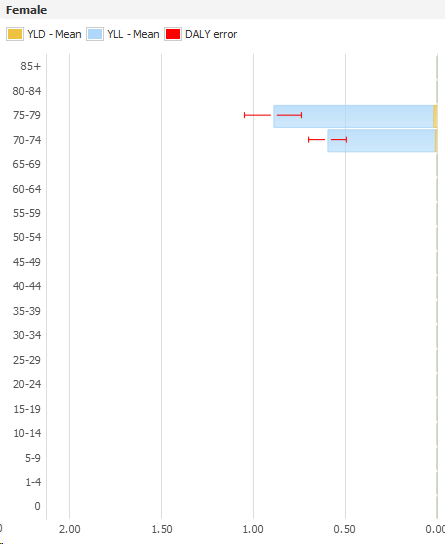

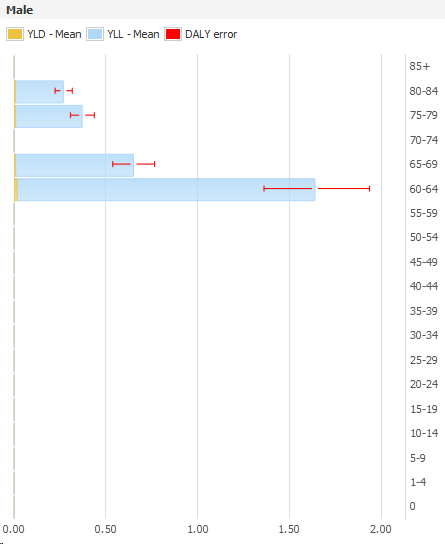


**Fig P. Sex- and age-group specific disease burden of shigellosis, from the BCoDE toolkit.**


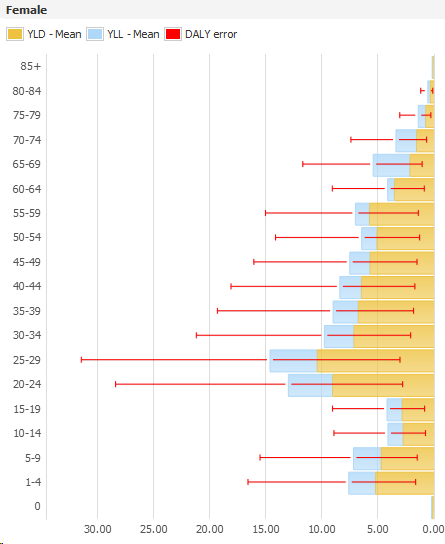

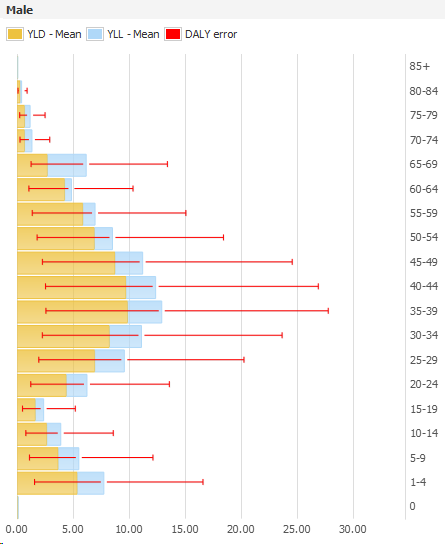


**Fig R. Sex- and age-group specific disease burden of vCreutzfeld-Jakob disease, from the BCoDE toolkit.**


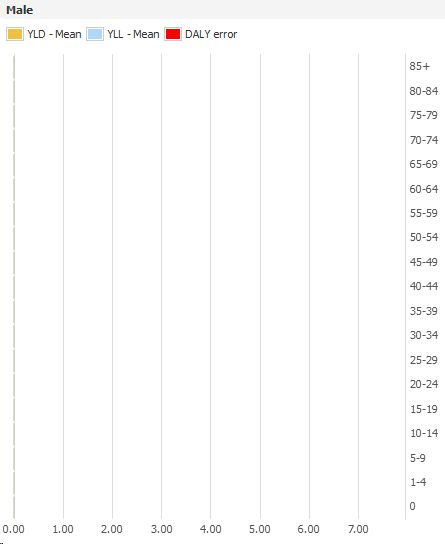

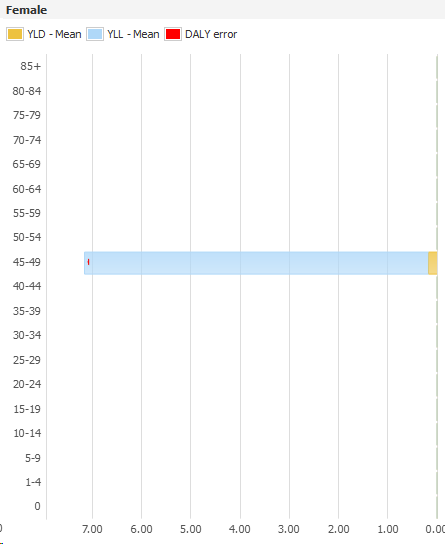


**Fig S. Sex- and age-group specific disease burden of influenza, from the BCoDE toolkit.**


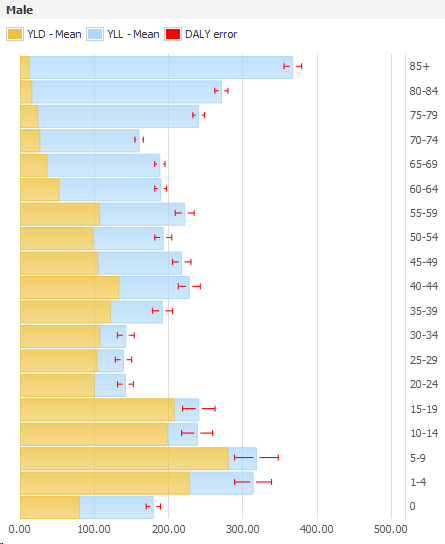

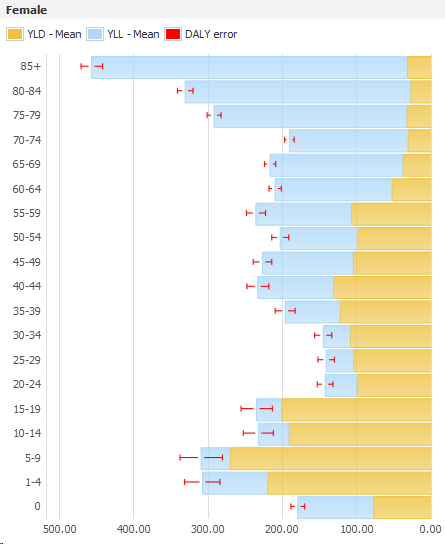


**Fig T. Sex- and age-group specific disease burden of legionellosis, from the BCoDE toolkit.**


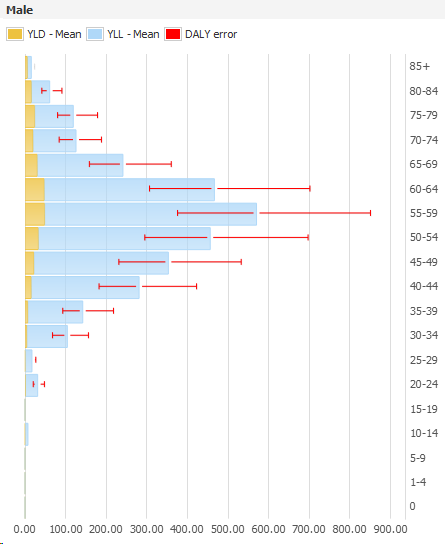

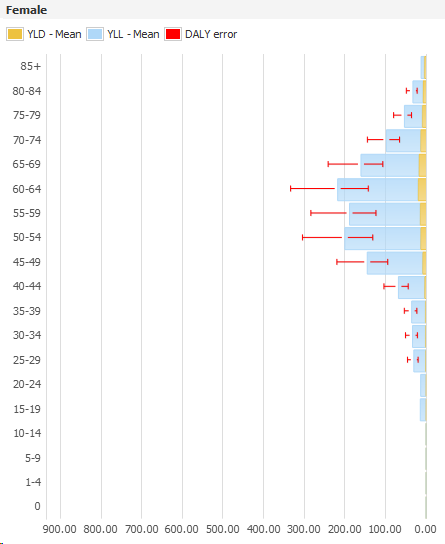


**Fig U.** **Sex- and age-group specific disease burden of Q fever, from the BCoDE toolkit.**


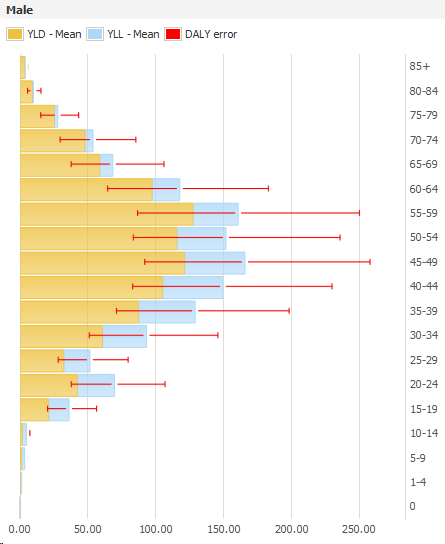

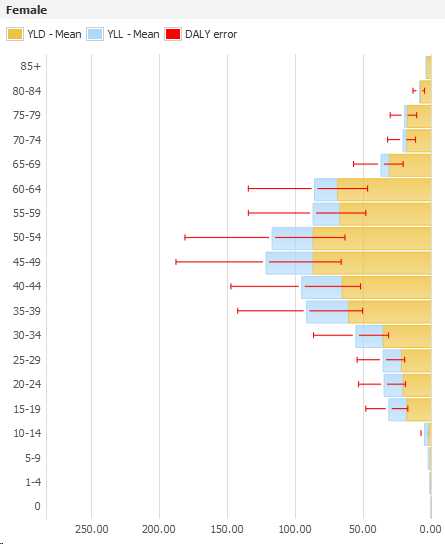


**Fig V. Sex- and age-group specific disease burden of tuberculosis, from the BCoDE toolkit.**


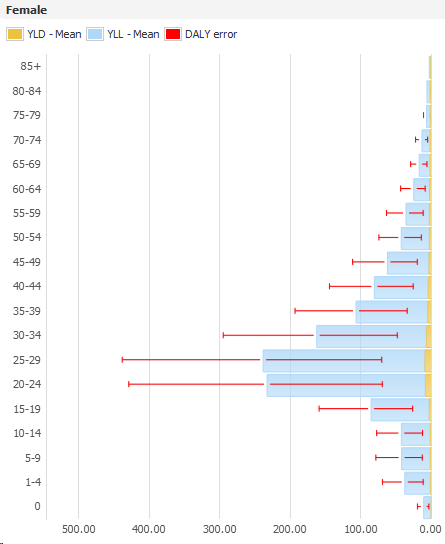

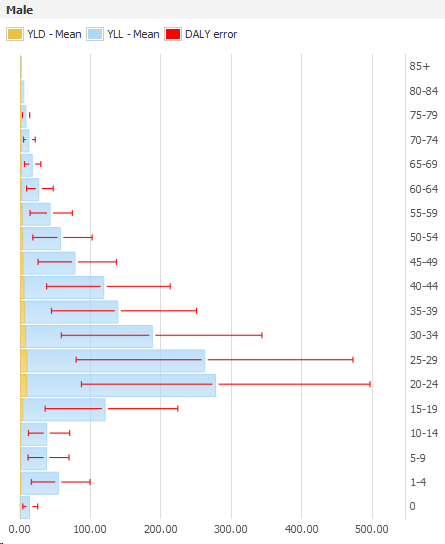

Supplement: S2 Appendix — (DOCX) [file pone.0153106.s002.docx]
